# TURBOMOLE: Today and Tomorrow

**DOI:** 10.1021/acs.jctc.3c00347

**Published:** 2023-06-29

**Authors:** Yannick J. Franzke, Christof Holzer, Josefine H. Andersen, Tomislav Begušić, Florian Bruder, Sonia Coriani, Fabio Della Sala, Eduardo Fabiano, Daniil A. Fedotov, Susanne Fürst, Sebastian Gillhuber, Robin Grotjahn, Martin Kaupp, Max Kehry, Marjan Krstić, Fabian Mack, Sourav Majumdar, Brian D. Nguyen, Shane M. Parker, Fabian Pauly, Ansgar Pausch, Eva Perlt, Gabriel S. Phun, Ahmadreza Rajabi, Dmitrij Rappoport, Bibek Samal, Tim Schrader, Manas Sharma, Enrico Tapavicza, Robert S. Treß, Vamsee Voora, Artur Wodyński, Jason M. Yu, Benedikt Zerulla, Filipp Furche, Christof Hättig, Marek Sierka, David P. Tew, Florian Weigend

**Affiliations:** † Fachbereich Chemie, Philipps-Universität Marburg, Hans-Meerwein-Str. 4, 35032 Marburg, Germany; ¶ Institute of Theoretical Solid State Physics, Karlsruhe Institute of Technology (KIT), Wolfgang-Gaede-Str. 1, 76131 Karlsruhe, Germany; § DTU Chemistry, Department of Chemistry, Technical University of Denmark, Kemitorvet Building 207, DK-2800 Kongens Lyngby, Denmark; ∥ Division of Chemistry and Chemical Engineering, California Institute of Technology, Pasadena, California 91125, United States; ⊥ Institute for Microelectronics and Microsystems (CNR-IMM), Via Monteroni, Campus Unisalento, 73100 Lecce, Italy; # Center for Biomolecular Nanotechnologies @UNILE, Istituto Italiano di Tecnologia, Via Barsanti, 73010 Arnesano, Italy; @ Institut für Chemie, Theoretische Chemie/Quantenchemie, Sekr. C7, Technische Universität Berlin, Straße des 17 Juni 135, 10623, Berlin, Germany; △ Institute of Inorganic Chemistry, Karlsruhe Institute of Technology (KIT), Engesserstr. 15, 76131 Karlsruhe, Germany; ∇ Department of Chemistry, University of California, Irvine, 1102 Natural Sciences II, Irvine, California 92697-2025, United States; ○ Institute of Physical Chemistry, Karlsruhe Institute of Technology (KIT), Fritz-Haber-Weg 2, 76131 Karlsruhe, Germany; ◆ Department of Chemistry, Case Western Reserve University, 10900 Euclid Ave, Cleveland, Ohio 44106 United States; ▲ Institute of Physics, University of Augsburg, Universitätsstr. 1, 86159 Augsburg, Germany; ● Otto-Schott-Institut für Materialforschung, Friedrich-Schiller-Universität Jena, Löbdergraben 32, 07743 Jena, Germany; ◊ Department of Chemical Sciences, Tata Institute of Fundamental Research, Homi Bhabha Road, Colaba, Mumbai 400005, India; ⧫ Department of Chemistry and Biochemistry, California State University, Long Beach, 1250 Bellflower Boulevard, Long Beach, California 90840-9507, United States; ⋈ Lehrstuhl für Theoretische Chemie, Ruhr-Universität Bochum, 44801 Bochum, Germany; ⧓ Institute of Nanotechnology, Karlsruhe Institute of Technology (KIT), Hermann-von-Helmholtz-Platz 1, 76344 Eggenstein-Leopoldshafen Germany; ⧖ Physical and Theoretical Chemistry Laboratory, University of Oxford, South Parks Road, Oxford OX1 3QZ, United Kingdom; ■ Institute of Chemistry, The Hebrew University of Jerusalem, Jerusalem 91904, Israel

## Abstract

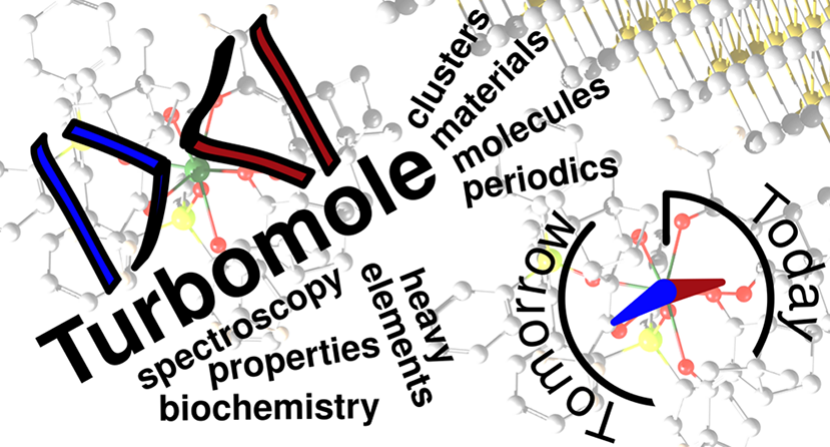

TURBOMOLE is a highly optimized software suite for large-scale quantum-chemical and materials science simulations of molecules, clusters, extended systems, and periodic solids. TURBOMOLE uses Gaussian basis sets and has been designed with robust and fast quantum-chemical applications in mind, ranging from homogeneous and heterogeneous catalysis to inorganic and organic chemistry and various types of spectroscopy, light–matter interactions, and biochemistry. This Perspective briefly surveys TURBOMOLE’s functionality and highlights recent developments that have taken place between 2020 and 2023, comprising new electronic structure methods for molecules and solids, previously unavailable molecular properties, embedding, and molecular dynamics approaches. Select features under development are reviewed to illustrate the continuous growth of the program suite, including nuclear electronic orbital methods, Hartree–Fock-based adiabatic connection models, simplified time-dependent density functional theory, relativistic effects and magnetic properties, and multiscale modeling of optical properties.

## 1 Introduction

TURBOMOLE is a collaborative, multinational software development project aiming to provide highly efficient and stable computational tools for quantum-chemical simulations of molecules, clusters, periodic systems, and solutions. The software suite is optimized for widely available, inexpensive, and resource-efficient hardware, such as multicore workstations and medium-size compute clusters. TURBOMOLE specializes in electronic structure methods with an outstanding accuracy–cost ratio, such as density functional theory including the random phase approximation (RPA), *GW*-Bethe–Salpeter equation (BSE) methods, second-order Møller–Plesset (MP2) theory, and coupled-cluster (CC) methods. The code is based on Gaussian basis sets and has been pivotal for the development of many fast and low-scaling algorithms in the past three decades, such as integral-direct methods, the resolution-of-the-identity (RI) approximation, fast multipole methods, imaginary frequency integration, Laplace transform, and pair natural orbital methods.

The development of TURBOMOLE was started in the late 1980s by Reinhart Ahlrichs and his group. Integral-direct algorithms and non-Abelian point group symmetry were among the first distinctive capabilities of TURBOMOLE, which initially focused on Hartree–Fock (HF) methods, with subsequent extensions for second-order MP2 perturbation theory^1−7^ and time-dependent HF (TDHF) response properties.^8^ A major milestone was the relatively early adoption of density functional theory (DFT) using newly designed quadrature algorithms^9^ and that of time-dependent DFT (TDDFT) shortly afterward.^10^ With extensions to meta-GGA,^11^ RPA, and other fifth-rung methods,^12−16^ as well as current density^17^ and local hybrid functionals,^18,19^ critical performance improvements,^20−24^ and a plethora of available analytical derivatives of ground- and excited-state energies,^25−39^ TURBOMOLE has become one of the leading all-purpose molecular (TD)DFT codes. The development and implementation of the RI approximation for Coulomb (RI-*J*^40,41^) and exchange contributions, (RI-*K*^42,43^), as well as its generalization to post-HF theories such as MP2 and CC2^44−46^ and its multipole-accelerated version for extended systems (MARI-*J*), were other critical innovations.^47^ The RI methods are still cornerstones in many modern implementations and outstanding features of the program suite.^23,31,48−68^ More recent additions include explicitly correlated wave function methods up to CCSD(T) and BCCD(T),^68,69^ efficient pair natural orbital (PNO) approaches,^70−73^ solvation models and embedding,^74−83^ two-component relativistic methods,^84−86^ GW-BSE type methods^52,87,88^ real-time (RT) TDDFT,^89^ and nonadiabatic molecular dynamics.^90^

To ensure continuity and coordinate the development, maintenance, and distribution independent of individual developers or groups, TURBOMOLE GmbH, a limited liability company located in Karlsruhe, Germany, was founded in 2007. TURBOMOLE GmbH has adopted an irrevocable bylaw preventing the distribution of dividends to ensure that all profits are reinvested into the project. TURBOMOLE GmbH distributes fee-based end-user licenses itself and through partners, as well as free developer licenses and access to the source code based on project proposals.^91^

Here we focus on recent developments and provide illustrative applications to chemistry and materials science. For an overview of existing features, as well as development, licensing, and distribution, the reader is referred to refs (92) and (93) and the TURBOMOLE Web site.^91^

## 2 Brief Feature Overview

The program suite consists of a series of modules with a broad range of methods from universal force field to fast semiempirical methods, state-of-the-art DFT and MP2, and coupled-cluster and post-HF methods for ground and excited states. For convenience, the use of modules is facilitated by various tools such as the scripts woelfling, raman, vcd, and genetic.py for reaction path optimization,^103^ vibrational Raman spectra,^29^ vibrational circular dichroism spectra (VCD),^104^ and genetic algorithms, respectively.^105^ Moreover, the graphical user interface TmoleX is of great help for running calculations and visualizing results.^106^

Almost all time-consuming parts are parallelized for multicore systems or clusters using OpenMP^107^ for shared-memory parallelization (SMP)^46,62,95−97^ and the message-passing interface^108^ (MPI) for parallelization across multiple nodes,^98−102^ as outlined in Table 1. The older Fork-SMP^94^ is available as a fallback for some modules. Starting with the latest release (V7.7), support for graphics processing units (GPUs) has become available.^109^

**Table 1:** Available Parallelizations of Various Modules Shown in Version 7.7^a^

module	functionality	Fork-SMP	OpenMP	MPI	OpenMP/MPI	GPU
dscf	HF/DFT energy	√	√	√	√	√
grad	HF/DFT gradient	√	√	√	√	√
ridft	RI-HF/RI-DFT energy	√	√	√	X	√
rdgrad	RI-HF/RI-DFT gradient	√	√	√	X	√
aoforce	HF/DFT Hessian	√	√	√	√	√
escf	HF/DFT/*GW*-BSE excitation energies	√	√	√	√	√
egrad	HF/DFT excited-state gradient	√	√	X	X	√
mpshift	NMR/EPR parameters (HF/DFT/MP2)	X	√	X	X	√
evib	electron transport (HF/DFT)	√	√	√	√	X
odft	orbital-dependent DFT energies	X	√	X	X	X
mpgrad	MP2 energy, gradient	√	X	√	X	X
ricc2	RI-MP2, ADC(2), CC2 energies, gradients, spectra	X	√	√	√	X
pnoccsd	PNO-MPPT and PNO–CC energies with F12	X	√	√	√	X
ccsdf12	CCSD, CCSD(T) energies with F12	X	√	X	X	X
rirpa	RPA energy, gradient	X	√	X	X	X
riper	periodic HF/DFT energy, gradient	X	√	X	X	X

a Fork-SMP^94^ and the OpenMP version^46,62,95−97^ are restricted to calculations on a single node. MPI^5,98−101^ and OpenMP/MPI hybrid^102^ implementations allow for the use of multiple nodes. The availability of first- and second-order derivatives as well as excitation energies is also indicated.

The list of parallelized HF/DFT modules includes those for molecular self-consistent field (SCF) energy (dscf and ridft) and gradient calculations (grad and rdgrad), response properties such as vibrational frequencies (aoforce), NMR/EPR spectra (mpshift), excited-state properties (escf, egrad), and electron transport properties (evib). For these modules, the OpenMP version is recommended for most calculations due to its cost–benefit ratio in terms of computer hardware. Accordingly, post-Kohn–Sham methods (rirpa) and calculations with periodic boundary conditions at the HF/DFT level (riper), as well as the CCSD and CCSD(T) program ccsdf12, are only parallelized with OpenMP. In contrast, the implementation of approximate CC methods in ricc2 and pnoccsd widely supports the OpenMP and MPI standard and combinations thereof for large-scale calculations on multiple nodes.

## 3 Recent Developments

### 3.1 Local Hybrid Functionals for Strong Correlation and Range-Separated Local Hybrid Functionals

Local hybrid functionals (LHs)^110,111^ with a position-dependent exact-exchange (EXX) admixture governed by a local mixing function (LMF) have been part of TURBOMOLE since release V7.2 and have since been extended in various ways over the past years. Using seminumerical integration techniques, such LHs have been implemented in an efficient way in the code,^18^ with functionalities that exceed by far those available in any other quantum-chemistry package that contains LHs. Beyond ground-state SCF^112^ and nuclear gradients,^33^ this now includes linear-response TDDFT energies^19,113^ and excited-state gradients,^35^ frequency-dependent and frequency-independent polarizabilities,^113,114^ NMR chemical shifts^115^ and spin–spin coupling constants,^60,86^ EPR hyperfine couplings and g-tensors,^116,117^ the related NMR shieldings of paramagnetic systems,^118^ magnetizabilities,^119^ and quasiparticle states based on *GW*.^119^ From the variety of possible applications and evaluations, many of which have been touched upon in the 2020 overview of TURBOMOLE^92^ and in a 2019 comprehensive review of local hybrids,^110^ we highlight in particular the outstanding performance of LHs for mixed-valence systems^120−122^ and for phosphorescence spectra.^123−125^ We also point to further recent LH publications and to reviews by other authors.^111,117,119,126−128^

Here, we focus on two recent extensions. Essentially, the aims of these works have been to conserve the established advantages of LHs and improve other aspects. We start with the fundamental goal to escape the often invoked “zero-sum game”^129,130^ between reduced self-interaction errors and delocalization errors or “fractional-charge errors” (FCEs) on one side and minimizing static-correlation errors or “fractional-spin errors” (FSEs) on the other side.^131^ The enhanced EXX admixture usually helps minimize FCEs, and LHs have been shown to achieve this goal while retaining some of the important left–right correlation in bonding regions.^110^ On the other hand, larger EXX admixtures usually are detrimental in cases with large FSEs, such as dissociating or stretched bonds or many transition-metal systems with appreciable static correlation. Standard LHs so far have not been a way out of this dilemma, at least not to a larger extent. Relevant real-space approaches to reduced FSEs are Becke’s B13 functional^132^ and a modified approach by Kong and Proynov (KP16/B13),^133^ which have both been implemented self-consistently into a local developer’s version of TURBOMOLE.^134^

**Figure 1:**
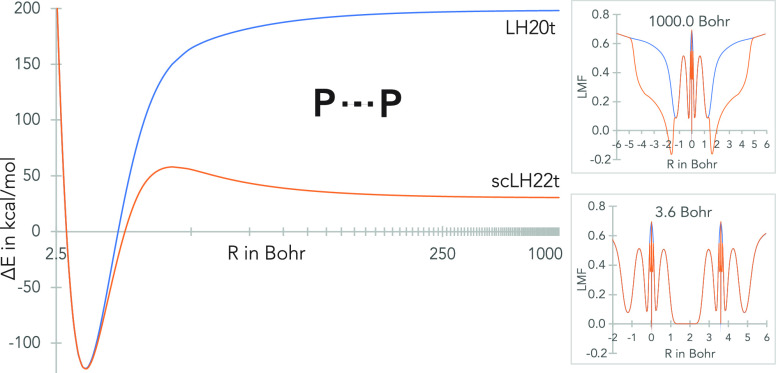
Spin-restricted dissociation curve of P_2_ with the original (LH20t) and sc-corrected t-LMF (scLH22t) along the bond axis for different distances on the dissociation curve (right). See ref (135) for related graphics for other molecules.

Circumventing the numerical difficulties and poor SCF convergence in many cases of the B13 and KP16/B13 functionals, the idea of a local strong-correlation factor has recently been transferred to the LH framework. Initial attempts were still based on relatively simple first-generation LHs but did already show that FSEs can be reduced when multiplying the LH term for nondynamical correlation (NDC) by a somewhat modified KP16/B13-type *q*_AC_ factor.^134^ Most recently, the more advanced scLH22t functional has been constructed.^135^ It is based on the more recent and overall more accurate LH20t functional.^120^ Using a damping factor for smaller NDC contributions, an almost complete decoupling between the underlying LH20t and the added *q*_AC_ factor could be obtained. That is, the optimized parameters of LH20t, as well as its excellent performance for weakly correlated situations (e.g., for GMTKN55 main-group energetics), are retained, but FSEs and the related spin-restricted dissociation curves of covalent bonds are dramatically improved.^135^ Notably, the *q*_AC_ factor forms part of a new LMF (Figure 1).

The second extension of LHs has been the implementation and construction of range-separated local hybrids (RSLHs), combining the ideas of local hybridization in real space and range-separated hybrids in interelectronic distance space.^136,137^ That is, instead of full-range semilocal exchange, short-range exchange is mixed in. This corresponds to the use of a long-range EXX admixture governed by a suitable range-separation parameter ω. Based on earlier work for locally range-separated hybrids,^138^ RSLHs have been implemented for ground-state SCF (modules dscf/ridft) and gradients (modules grad/rdgrad), as well as for linear-response TDDFT (module escf).^139^ Using this implementation, the ωLH22t RSLH has been constructed and optimized. It retains most of the good performance of LH20t for main-group and transition-metal energetics, as well as for core, Rydberg, and triplet valence excitations. At the same time, however, it decisively corrects errors for excitations with appreciable charge-transfer character and improves on the potential-energy curves of three-electron cations known to be affected strongly by self-interaction errors.^139^ Most recently, ωLH22t has been demonstrated to provide unprecedented accuracy in quasiparticle energies for applications in molecular electronics and organic photovoltaics, without the usual system-dependent tuning of range-separated hybrids.^140^

### 3.2 Inclusion of the Current Density in DFT

The kinetic energy density τ(**r**) is a commonly used ingredient in many functionals to detect iso-orbital regions or describe the inhomogeneity of the electron density ρ(**r**). For its extension τ(**r**,*t*), used in the time-dependent Kohn–Sham formalism (TDKS), it has been shown^17^ that this quantity is not invariant under a gauge transformation in the external potential. Substitution of τ by its generalization^141−143^, where **j**_P_ is the paramagnetic current density, restores gauge invariance. This leads to additional terms in the magnetic orbital rotation Hessian in linear-response TDDFT calculations, accounting for the response of **j**_P_. While the original implementation of these terms in TURBOMOLE dates back to 2012 (release V6.4), we highlight four recent important updates.

First, an incorrect prefactor 2 has been removed from the original implementation with V7.6.^119,144^ TDDFT calculations employing τ-dependent functionals performed with previous versions erroneously overcorrected for the effects of the current density response and should be reassessed, although average changes are on the order of 0.03 eV.^144^

**Figure 2:**
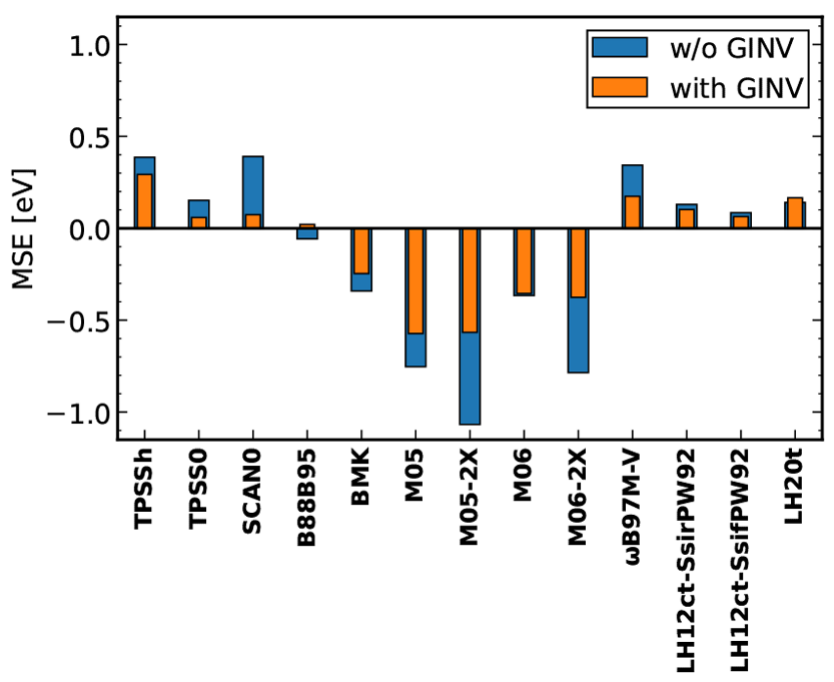
Effect of restoring gauge invariance (GINV) for various τ-dependent functionals on the average deviation of vertical TDDFT excitation energies from the experimental absorption maxima for five Ni(II) complexes.^146^ Reprinted from ref (145) with permission. Copyright 2022 AIP Publishing.

Second, a recent investigation^145^ reveals that the effect restoring gauge invariance has on the final excitation energies can be significantly larger than previously assumed depending on the functional and type of excitation. In one particular investigation of d–d excitations in nickel(II) complexes, restoring gauge invariance shifts the excitation energies with the M06-2X functional by more than 0.4 eV closer to the experimental reference values, as shown in Figure 2.^145^

A broader analysis reveals that the importance of imposing gauge invariance can be linked to the derivative of the exchange energy integrand with respect to τ.^145^ Moreover, *n* → π* excitations are significantly more affected by restoring gauge invariance than most π → π* excitations with the exception of π → π_⊥_^*^ excitations, where the dominantly contributing molecular orbitals (MOs) are perpendicular (⊥) to each other.^145^ These findings suggest that a reassessment of previously reported TDDFT results obtained with τ-dependent functionals is warranted, particularly for cases that are potentially more sensitive due to the choice of the functional, the type of excitation, or both. Gauge invariance is restored by default with τ-dependent functionals at moderate (nonhybrid functionals) or negligible (hybrid functionals) additional computational cost.^17^ Recently, excited-state gradients and quadratic response properties for τ̂-dependent meta-generalized gradient approximations (mGGAs) have been implemented, enabling gauge invariant computations of excited-state equilibrium structures, relaxed dipole moments, (dynamic) hyperpolarizabilities, and two-photon absorption cross sections.^147^ These developments will be available in a future TURBOMOLE release.

**Figure 3:**
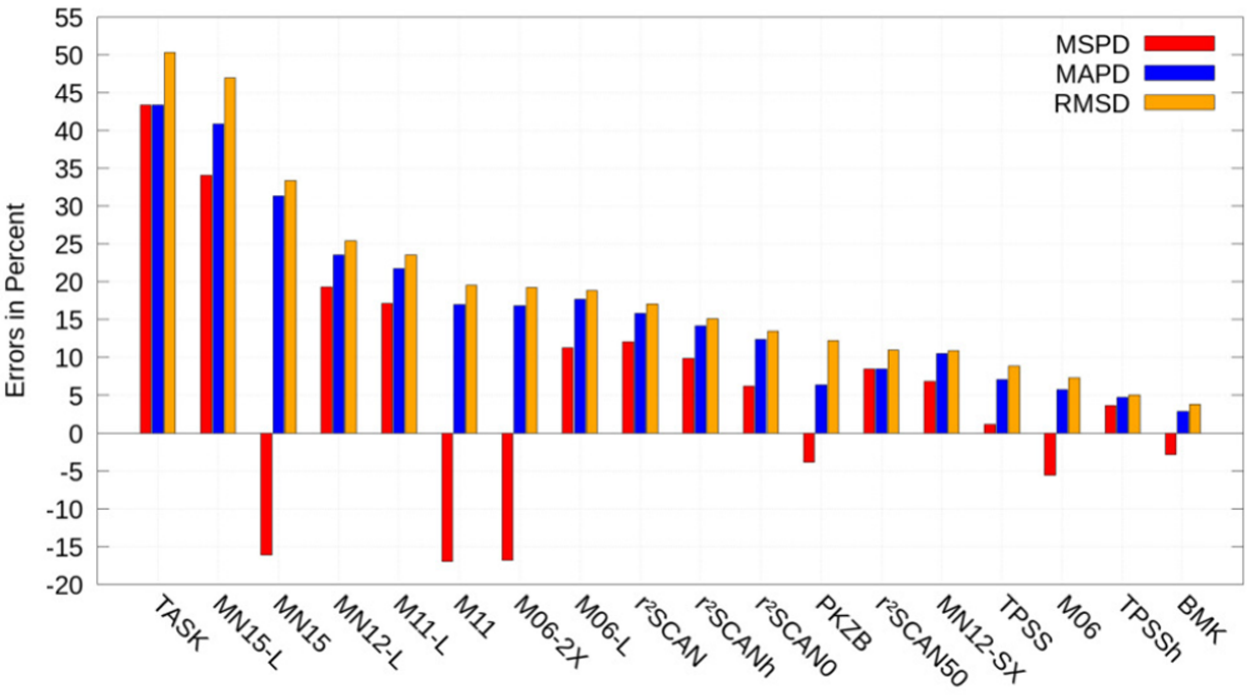
Impact of the current-dependent generalization of τ for various density functional approximations on the isotropic Δ*g*-shift of [MoNCl_4_]^2–^, [MoOF_4_]^−^, [MoOCl_4_]^−^, [MoOF_5_]^2–^, [MoOBr_5_]^2–^, [TcNF_4_]^−^, [TcNCl_4_]^−^, and [TcNBr_4_]^−^. Results with the well-known field-dependent generalization serve as reference.^150^ We list the mean signed percentwise deviation (MSPD), the mean absolute percentwise deviation (MAPD), and the root-mean-square deviation (RMSD). Reprinted from ref (118) under a CC BY license. Copyright 2022 the Authors.

Third, the inclusion of **j**_P_ is required for gauge invariance of magnetic properties and implemented for magnetizabilities,^119^ NMR coupling constants,^119^ and NMR shifts of closed-shell^119,148^ and open-shell systems,^118^ as well as EPR hyperfine coupling constants^149^ and g-tensors.^118^ Recent findings indeed hint at the inclusion of the current density response also being crucial for NMR and EPR properties.^118,119,148^ Especially for open-shell systems, neglecting the current density leads to large deviations,^118^ as shown in Figure 3.

Finally, it was shown that the inclusion of the paramagnetic current is also crucial in relativistic two-component calculations.^151^ Contrary to all previous cases, the modifications outlined above must already be taken into account for the ground state in the presence of spin–orbit coupling (SOC). SOC gives rise to an internal magnetic field, inducing a paramagnetic current that already has a nonvanishing contribution at the energy-level.^151^ Accordingly, if properties such as light–matter interactions are targeted in the presence of SOC, the interplay of the ground- and excited-state paramagnetic currents must be taken into account. This gives rise to highly nonlinear **j**_P_-dependent terms, which have a profound impact on many properties.^151,152^ Current density functional theory (CDFT) for τ-based functionals is available for ground-state energies (module ridft) and gradients (module rdgrad) and linear response properties (modules escf and mpshift).^151^ Given the profound impact of **j**_P_, we therefore strongly recommend the exclusive use of the current-dependent forms.

### 3.3 Methods for Finite Magnetic Fields

Quantum-chemical calculations are routinely carried out for various types of molecular properties in magnetic fields, including NMR, EPR, and magnetic circular dichroism (MCD) spectroscopy (see also sections 3.4, 3.5, 3.6, and 3.9.2 of this Review).^153−155^ For these applications, the magnetic field is usually treated perturbatively, as it is orders of magnitude smaller than the electronic interactions responsible for the formation of a chemical bond.

Other applications necessitate the use of a more general approach, particularly if the magnetic field becomes strong enough to compete with the electronic interactions within a molecule (>1000 T).^156−163^ Such conditions may be found in the vicinity of interstellar objects like magnetic white dwarfs and cannot be reproduced in a laboratory.^164−167^ Consequently, spectra obtained from such interstellar objects can only be interpreted using quantum-chemical calculations^168−172^ In such extreme conditions, entirely new types of chemical bonding, spin-phase transitions, and other exotic phenomena have been shown to occur.^162,173,174^ The effects of arbitrarily weak or strong magnetic fields on atoms or molecules may be computed using the finite magnetic field approach, which is implemented for Hartree–Fock,^175^ CDFT,^152,176^*GW*/BSE,^177,178^ RPA,^179^ and CC2.^178^ Through the calculation of electronic ground states, molecular gradients, and electronic excitations, a wide variety of applications for molecular spectroscopy in magnetic fields are now accessible.

**Figure 4:**
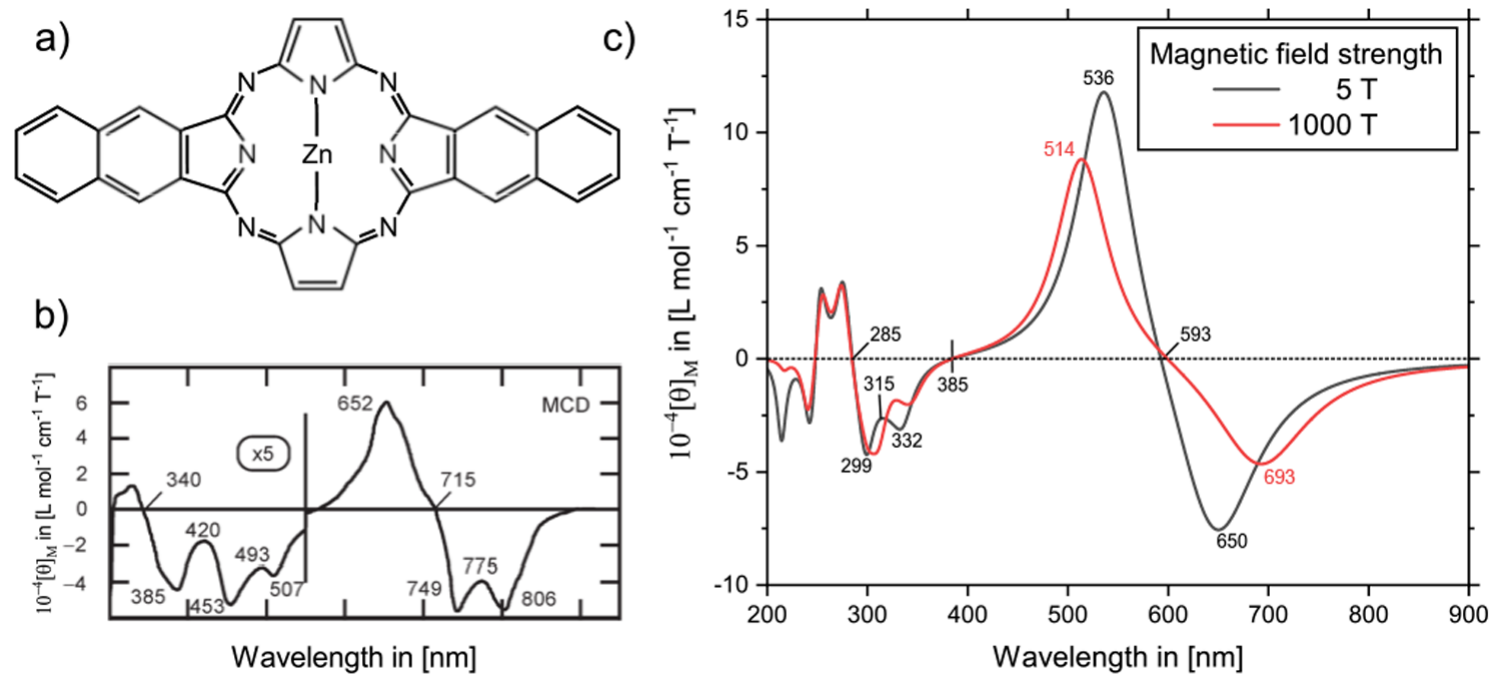
MCD spectrum of ZnDiNTAP. (a) Molecular structure. (b) Experimental spectrum. Reprinted with permission from ref (180). Copyright 2007 RSC Publishing. (c) Spectra as calculated in finite magnetic fields of 5 and 1000 T. Adapted with permission from ref (176). Copyright 2022 the Authors.

Due to the efficiency of our implementation, systems containing dozens of atoms can be routinely computed in explicit magnetic fields.^152,175,176,178^ To highlight the capabilities of our approach, we calculated the MCD spectrum of ZnDiNTAP,^176,180^ a zinc tetraazaporphyrin with two fused naphthalene units, Figure 4a, using CAM-B3LYP and a mixed def2-TZVP (Zn)/def2-SVP (all other atoms) basis set. Similarly to the experiment,^180^Figure 4b, a magnetic field of 5 T was applied. The resulting spectrum is shown in Figure 4c. Minor differences can all be attributed to solvation effects and vibronic coupling, which were neglected in our calculation.^176^ Furthermore, the MCD spectrum of ZnDiNTAP in an explicit magnetic field of 1000 T is shown in Figure 4c. While some of the bands, particularly in the fingerprint region, are not affected by nonlinear effects induced via such a strong field, the two Q bands are significantly shifted (650 → 693 nm and 536 → 514 nm).

Other applications of the finite magnetic field ansatz include higher-order properties such as magnetizabilites and hypermagnetizabilities through the use of numerical derivatives. Moreover, molecules that are naturally prone to exhibiting “magnetic” effects, such as aromatic compounds (see also section 3.7), may show a nonlinear response even to weak magnetic fields.^152^ This is not captured by a perturbative approach but can be routinely investigated using our implementation.

### 3.4 EPR Properties and Single-Molecule Magnets

Over the past decade, TURBOMOLE has pioneered the *in silico* study of f-element chemistry. To provide accurate descriptions of compounds containing these heavy elements, robust DFT routines are leveraged to deliver a balanced treatment of dynamic correlation, static correlation, solvation, and relativistic effects. More recently, these developments have enabled the discovery of new species with novel chemistry, subsequently necessitating new and improved computational methods capable of describing them.

For example, a series of recently discovered Ln-based single-molecule magnets (SMMs), [La(OAr*)_3_]^−^, [Lu(NR_2_)_3_]^−^, and [Lu(OAr*)_3_]^−^ (OAr* = 2,6-Ad_2_-4-*t*-Bu-C_6_H_2_O, Ad = adamantyl, *t*-Bu= *tert*-butyl, R = SiMe_3_ with Me = methyl), were found to exhibit exotic EPR properties, with [Lu(OAr*)_3_]^−^ producing hyperfine coupling (HFC) constants of unforeseen magnitude and furthermore demonstrating extended magnetic coherence facilitated by a hyperfine clock transition. A primary investigation of these species with nonrelativistic HFC operators attributed the large hyperfine coupling constants to a sizable Fermi contact contribution from the highest occupied MO (HOMO) of each system. However, the predictions of hyperfine coupling constants and g-tensor values themselves produced errors of roughly one order of magnitude, strongly suggesting the need for more rigorous methods.^181^

**Figure 5:**
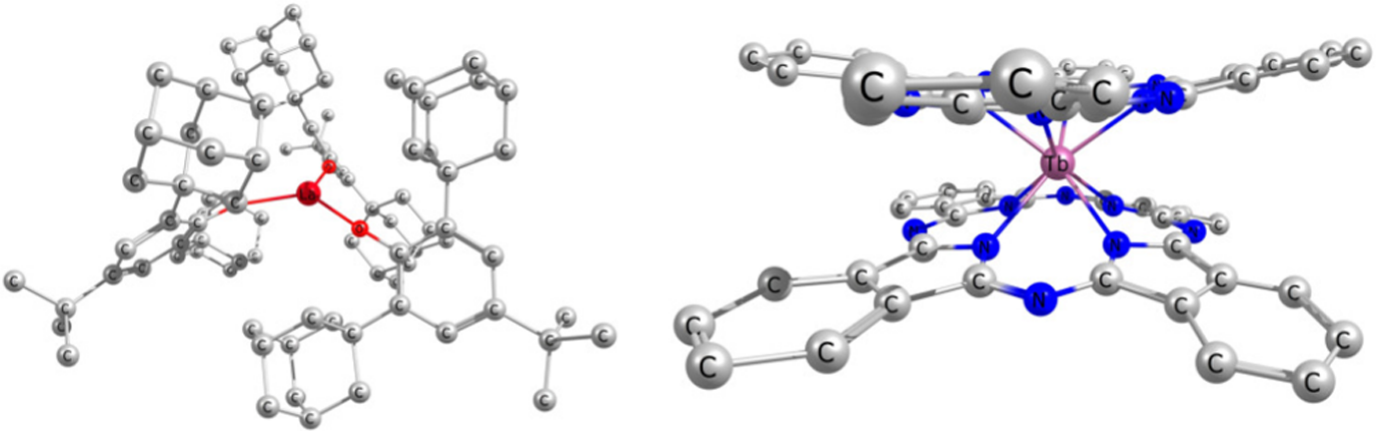
Molecular structure of single-molecule magnets [Lu(OAr*)_3_]^−^ and [TbPc_2_]^−^. Reprinted with permission from ref (151) under a CC BY license. Copyright 2022 the Authors.

Such improved predictions of EPR spectra are possible with relativistic exact two-component (X2C) theory,^38,39^ including spin–orbit effects up to the noncollinear two-component (2c) DFT framework.^38,39,117^ For meta-generalized gradient approximations and local hybrid functionals, this also includes the paramagnetic current density in the ground state (cf. section 3.2).^151^ This rigorous formulation can be truncated to the scalar-relativistic limit^63^ or a perturbative ansatz^149^ to study the individual contributions of each term to the EPR parameters. Gauge origin invariance of the *g*-tensor calculations is ensured by the gauge including atomic orbitals,^36,39,149^ which are crucial for systems with a spatially distributed spin density. These methods were implemented, and their performance is shown here for the aforementioned SMM [Lu(OAr*)_3_]^−^ shown in Figure 5. The all-electron relativistic methods lead to good agreement with the experiment, as shown in Table 2. The HFC constant of [Lu(OAr*)_3_]^−^ is dominated by the scalar-relativistic contribution due to the localization of the spin-density in the 6s/d HOMO producing a large Fermi contact interaction. The importance of the paramagnetic spin–orbit contribution increases with the number of unpaired electrons and the scalar formulation, as well as the spin–orbit perturbation theory (SOPT) break down for systems such as [TbPc_2_]^−^ with six unpaired electrons, Pc = bis(phthalocyaninato).^38,39,117,151^ The self-consistent 2c methods are thus pivotal.

**Table 2:** Principal Components for the HFC *A* of [Lu(OAr*)_3_]^−^ and Isotropic HFC *A* of [TbPc_2_]^−^ at Various Levels of theory^38,39,117,149^ and Comparison with Experimental Results (expt.)^181,182^^a^

		[Lu(OAr*)_3_]^−^			[TbPc_2_]^−^
method		*A*_*xx*_	*A*_*yy*_	*A*_*zz*_	*A*
TPSS	SR	3192	3192	3148	190
cTPSS	SO	3190	3190	3153	375
ωB97X-D	SR	3489	3489	3456	136
ωB97X-D	SO	3464	3464	3448	488
TMHF	SR	3225	3225	3171	293
cTMHF	SO	3208	3208	3161	526
Expt.		3500	3500	3400	519

a HFC is given in MHz. SR and SO denote scalar and spin–orbit relativistic results (x2c-TZVPall-2c/x2c-SVPall-2c basis), respectively.

With the next release version, the EPR Euler transformations for the HFC, *g*-tensor, and electric-field gradient as well as the nuclear quadrupole interaction tensor will further become available for users.^39^

### 3.5 NMR Coupling Constants Across the Periodic Table of Elements

NMR spectroscopy is key to the analysis and structure determination not only for organic compounds but also for inorganic systems consisting of heavy elements. The NMR spin–spin coupling constant describes the splitting of the signals or peaks in the NMR spectra and is a characteristic property driven by the chemical environment. Formally, the coupling tensor is obtained as the mixed derivative of the energy with respect to the corresponding nuclear magnetic moments, which are introduced via the principle of minimal coupling. NMR couplings are available within a nonrelativistic^60^ scalar X2C^183^ and the spin–orbit X2C framework.^86^ All functional classes up to local hybrids are supported and include the current density for gauge invariance.^61,119,151^

For systems made up of light elements, the nonrelativistic treatment is sufficient. Here, the coupling constant is generally partitioned into the Fermi-contact (FC), spin–dipole (SD), paramagnetic spin–orbit (PSO), and diamagnetic spin–orbit (DSO) contributions. The FC, SD, and PSO terms necessitate the solution of the response equations, whereas the DSO term is computed with the ground-state density. Typically, the FC contribution is the leading term, and accurate coupling constants require large basis sets. Thus, a nuclear selection scheme and locally dense basis sets are often applied to large-scale calculations.

**Figure 6:**
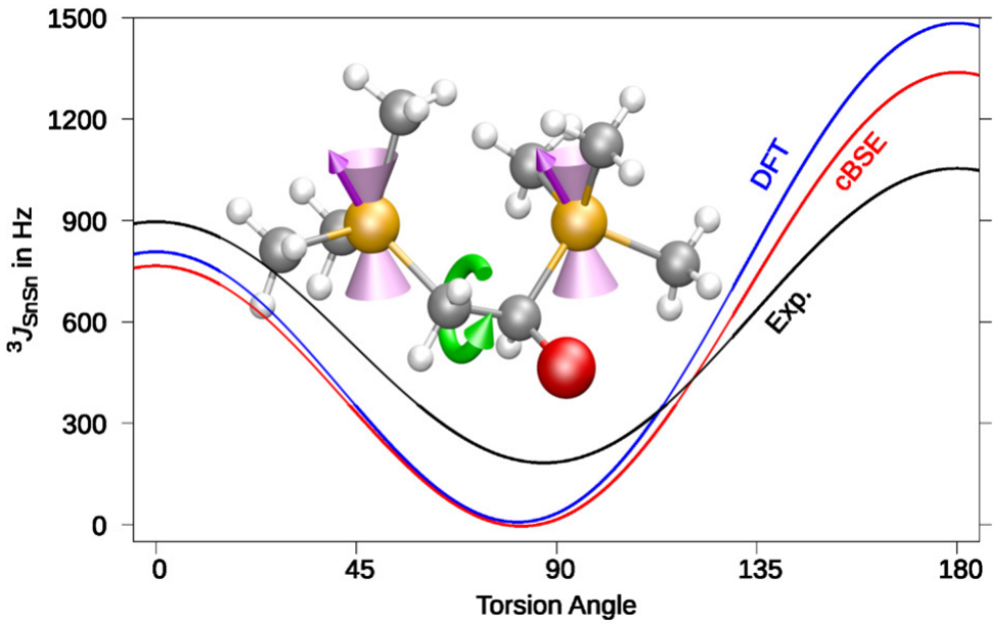
Fitted Karplus equation for Sn compounds: ^3^*J* = *A*cos(2ϕ) + *B*cos(ϕ) + *C*. For each Sn–Sn torsion angle ϕ, the average of the ^3^*J*_SnSn_ coupling constant over 13 compounds is computed. Blue, BH&HLYP/x2c-TZVPall-2c; red, *GW*-cBSE@BH&HLYP; and Exp., experimental findings.^184^ Adapted with permission from ref (61). Copyright 2022 the Authors.

Systems containing heavy elements such as Sn, Pb, Pd, and Pt require the inclusion of relativistic effects,^61,86,183,185−187^*i.e.*, methods based on the Dirac equation are introduced. For such methods, the FC, SD, and PSO terms are coupled due to spin–orbit interaction, and they come with drastically increased computational demands. Nevertheless, when using a local X2C ansatz,^84−86^ large-scale calculations are possible, as illustrated in Figure 6 for the Karplus relationship of Me_3_Sn–CH_2_–CHR–SnMe_3_, where R is different substituents (Me = CH_3_). The relativistic DFT approach reproduces the experimental findings with fairly good agreement. Improvements are possible with the correlation kernel augmented BSE (cBSE) based on the Green’s function *GW* ansatz. Here, the DFT response equations are replaced with their BSE counter parts.^61,113^

To demonstrate the efficiency, the calculation of the Sn–P coupling constants of [({SIDipp}P)_2_Sn] (SIDipp = 1,3-bis(2,6-di-isopropylphenyl)-imidazolidin-2-ylidene) with 137 atoms^187^ takes about 44 min (PBE) and 55 h (PBE0) using 12 OpenMP threads of an Intel Xeon Gold 6212U CPU (2.40 GHz).^86^ Notably, using a single NVIDIA A100 GPU, the PBE0 timing can be reduced to 3.5 h.

### 3.6 Paramagnetic NMR Shieldings and Shifts

**Figure 7:**
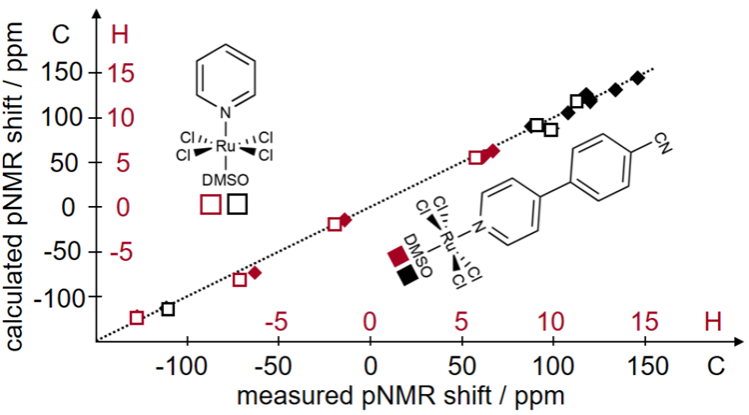
Computational results (ωB97X-D/x2c-TZVPall-s) and experimental findings for two negatively charged Ru(III) compounds. Adapted with permission from ref (149). Copyright 2022 the Authors.

NMR spectroscopy is also an important technique for the characterization of open-shell chemical compounds. NMR shielding tensors and chemical shifts describe the positions of the peaks in NMR spectra. In the closed-shell case, only the temperature-independent orbital contribution is relevant to the calculation of the shielding tensor. Both a nonrelativistic treatment^3,62,188^ and a scalar-relativistic treatment^36,104^ are available for the orbital contribution, including the response of the current density.^118,119,148^ The paramagnetic NMR (pNMR) shielding tensor **σ**_*I*_^tot^ for a nucleus *I* reads
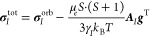
1with *S* denoting the spin, μ_*e*_ denoting the Bohr magneton, γ_*I*_ denoting the gyromagnetic ratio of nucleus *I*, *k*_B_ denoting the Boltzmann constant, and *T* denoting the temperature. Here, the orbital contribution **σ**^orb^ is the straightforward open-shell generalization of the closed-shell limit.^63,118^ Additionally, a temperature-dependent contribution arises, which includes the HFC tensor *A*_*I*_ of nucleus *I* and the *g*-tensor *g* already discussed in section 3.4. Both the HFC and the *g*-tensor depend on spin–orbit coupling. For the calculation of ^1^H/^13^C pNMR spectra of large molecules, a perturbative treatment of spin–orbit coupling is preferred over the 2c ansätze due to lower computational costs.^149^ The viability of the pertubative ansatz in the X2C framework is demonstrated for two negatively charged Ru(III) complexes in Figure 7, which depicts the good agreement between calculated results and the experimentally measured^189^ pNMR ^1^H and ^13^C shifts of the two compounds.

The calculation of properties depending on the density in the vicinity of the nuclei requires additional tight basis functions. Thus, the pcJ,^190^ pcS,^191^ and pcH^192^ basis sets are recommended for nonrelativistic calculations. For relativistic calculations, the x2c-s basis sets were developed.^193,194^

**Figure 8:**
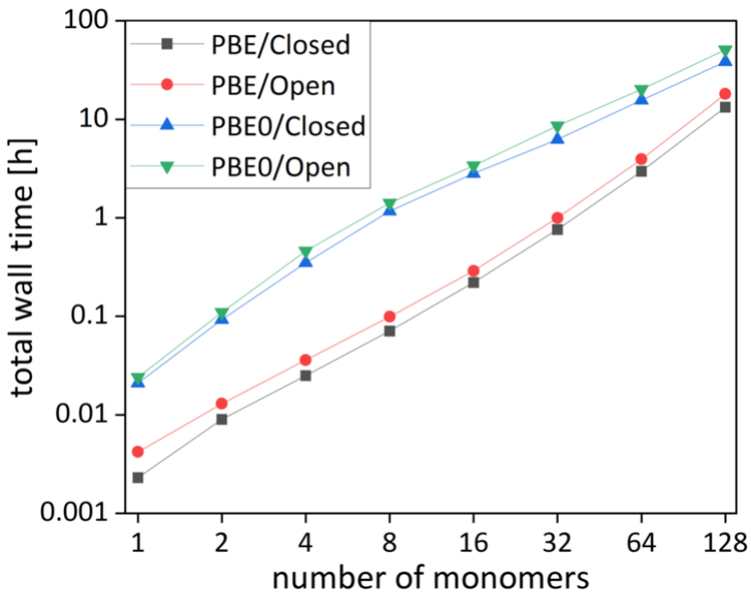
Total wall times for SCF and NMR calculations with respect to the number of glucose units (6-31G* basis). Wall times were measured on a single thread of an Intel Xeon Gold 6212U CPU (2.40 GHz). Reprinted with permission from ref (63). Copyright 2021 the Authors.

The efficiency of the pNMR implementation is similar to that of the closed-shell case, as shown in Figure 8. Coulomb integrals can be calculated with the RI-*J*/MARI-*J* approximations for the Coulomb contribution^62,63^ and the seminumerical scheme for exchange integrals.^18,109^

### 3.7 Ring Currents of Heavy-Metal Clusters

Aromatic compounds, such as benzene, show characteristic signals at 7 ppm in the ^1^H NMR spectra. This shift is a consequence of the cyclic electron delocalization associated with the π-orbitals, which deshield the nuclei due to an induced ring current.^195^ This magnetically induced current density may be calculated indirectly with the nucleus-independent chemical shift^196^ (NICS) or directly using TURBOMOLE’s interface to the GIMIC program, which was reworked for release V7.7 and now supports open-shell calculations.^197−200^ The latter approach is more flexible and also applicable to complicated multicyclic systems.^201−204^

**Figure 9:**
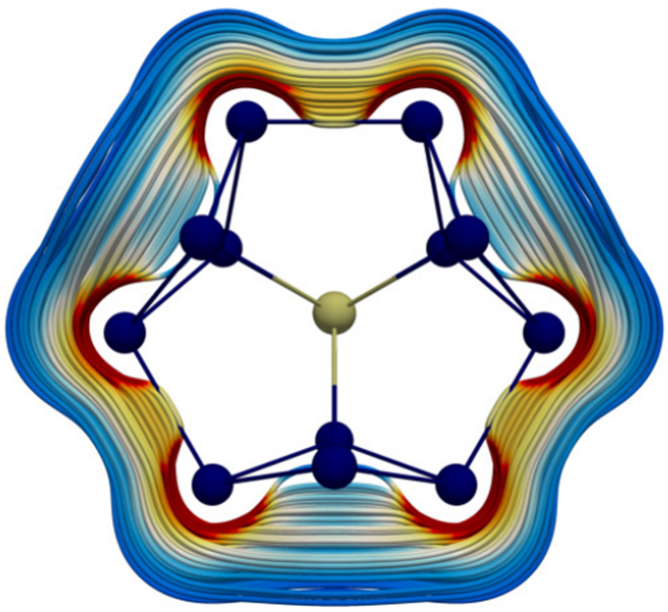
Aromatic ring current of [Th@Bi_12_]^4–^. The color scheme (red to blue) indicates strong to weak currents. Data from ref (205)

The occurrence of ring currents and the concept of aromaticity are not restricted to cyclic conjugated hydrocarbons and related organic compounds. All-metal systems may also sustain a ring current, and these systems are therefore classified as all-metal aromatic compounds. For instance, the endohedral [Th@Bi_12_]^4–^ cluster features a nonlocalizable π-orbital around the {Bi_12_} torus, which leads to a ring current.^205^Figure 9 shows a streamlined representation of this ring current, whose strength amounts to about 25 nA/T.^119,205^

Despite featuring only two delocalized π-electrons, almost the same ring current strength as that in porphine is induced. Here, the thorium atom in the torus center is only needed for stability and the synthesis, but it does not take part in the ring current. Furthermore, the open-shell variant [U@Bi_12_]^3–^ shows a strong ring current,^63^ as the same π-orbital is occupied by two electrons.^205,206^ Moreover, prismatic {Bi_6_}-based clusters such as [(CpRu)_3_Bi_6_]^−^ show ring currents of more than 25 nA/T.^207^ Therefore, all-metal clusters help to push the frontiers of aromaticity.

### 3.8 Characterization of Novel Electronic Configurations in f-Block Elements by DFT and RPA Methods

Electronic structure calculations of large f-element complexes contain a great deal of complexity due to the competition between metal oxidation states, d- and f-shell occupations, spin coupling, and relativistic effects.^208^ Computational studies in these compounds have to scan a range of formal electronic occupations and apply a range of techniques to ensure convergence to the desired electronic configuration, including Fermi smearing with suitable parameters,^209,210^ and a combination of damping, level shifting,^211^ and direct inversion in the iterative subspace (DIIS) extrapolation.^212^ Moreover, the stability of the ground-state reference is a concern in calculations of molecular properties, for example, electronic absorption spectra.^213^

DFT results helped characterize the structures and properties of nontraditional Ln^2+^ complexes possessing the 4f^*n*^5d^1^ configuration in the [Ln(C_5_H_4_SiMe_3_)_3_]^−^ (Ln = Ce–Nd, Gd–Er),^214−217^ [Ln{N(SiMe_3_)_2_}_3_]^−^ (Ln = La, Gd),^218,219^ and [Ln(Cp^iPr_5_^)_2_] (Ln = Tb, Dy) series.^220^ The preference for the 4f^*n*^5d^1^ configuration relative to the traditional 4f^*n*+1^ occupation of Ln^2+^ results from the stabilization of the Ln 5d_*z*^2^_ orbital by the trigonal ligand environment or extremely bulky ligands. Nontraditional Ln^2+^ complexes show a characteristic intense absorption band in the visible range due to excitations from the occupied Ln 5d orbital. The prediction of UV–vis spectra of low-valent lanthanide complexes, in particular those with a nontraditional configuration, is improved by including diffuse augmentation in lanthanide basis sets.^221^ DFT and RPA methods were employed to examine the strong ferromagnetic coupling between the Ln^3+^ centers in [(C_5_Me_5_)_2_Ln(μ-S)_2_Mo(μ-S)_2_Ln(C_5_Me_5_)_2_]^−^ (Ln = Y, Gd, Tb, Dy) and the Mo→Ln electronic excitations in the near-infrared spectral region.^222^ Excited-state studies of [Ln(C_5_Me_5_)_2_(C_5_Me_4_H)] and [Ln(C_5_Me_5_)_2_(η^3^-C_3_H_4_)] complexes (Ln = Y, Dy, Lu) using TDDFT elucidated their unexpected photochemical activation, which was used to reduce dinitrogen and sulfur and to polymerize isoprene.^223,224^

**Figure 10:**
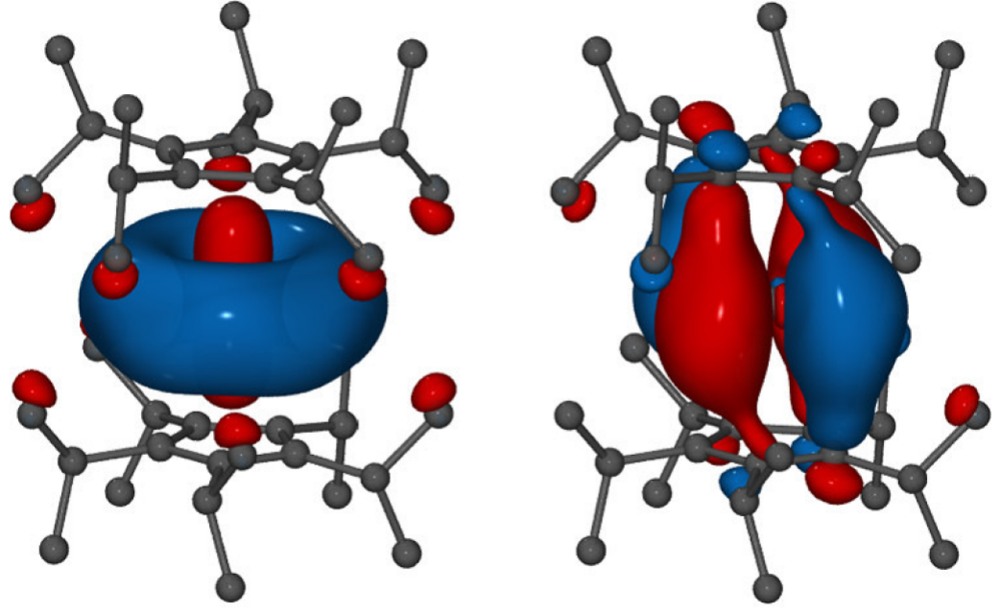
Contour plots of the HOMO (left) and LUMO (right) of [An(Cp^iPr_5_^)_2_] determined with TPSS,^226^ Stuttgart–Cologne scalar relativistic ECPs,^227^ and the corresponding basis sets.^228,229^ Orbital isovalues of 0.03 were used. Reprinted with permission from ref (225). Copyright 2019 American Chemical Society.

Computational studies of neutral actinide complexes [An(Cp^iPr_5_^)_2_] (An = Th, U, Pu, Am, Bk, No, Lr) (pentaisopropylcyclopentadienyl = Cp^iPr_5_^) using DFT predicted ground states with a linear ligand arrangement of *S*_10_ symmetry and significant An 6d orbital occupation for An = Th, U, Lr.^225^ The calculations were carried out with the TPSS exchange–correlation functional,^226^ Stuttgart–Cologne scalar-relativistic small-core effective core potentials (ECPs),^227^ and the corresponding basis sets.^228,229^ Mixed 5f/6d occupation was predicted in the corresponding Pu complex, while the An = Am, Bk, No complexes were found to have 5f^*n*+1^ configurations. The Pu and Am complexes showed a slight deviation from the perfectly symmetric structure, with Cp–M–Cp bending angles of 11° and 12°, respectively. The simulated absorption spectra showed intense peaks in the UV–vis range due to the metal–ligand charge transfer excitations from the An 6d orbital shown in Figure 10. Comparisons with the previously experimentally known Ln analogs (Ln = Dy, Tb)^220^ suggested that the synthesis of the predicted actinocene complexes was thermodynamically feasible. The computational predictions received experimental confirmation for An = U while the results were still under review. Layfield and co-workers reported the synthesis of the linear *S*_10_-symmetric “second-generation” uranocene [U(Cp^iPr_5_^)_2_].^230^ The U–Cp centroid distance was determined from crystallographic studies as 2.504 Å, in good agreement with the computational result of 2.483 Å. The measured UV–vis spectra showed broad and intense absorption, as predicted by TDDFT calculations.

### 3.9 Damped Response Theory

Assessing light–matter interactions in extended or exotic systems is an active field of development in TURBOMOLE.^53,119,152,176,178,231,232^ While root-by-root linear-response methods have been crucial for many applications, they are not suitable for spectrally dense systems or core excitation due to the high number of excited states (roots). Damped response theory,^233,234^ or the equivalent complex polarization propagator approach,^235^ provides a convenient framework to formulate resonance convergent response functions, circumventing these problems. It provides a convenient route to directly compute a variety of absorptive and dispersive effects in both UV–vis and X-ray frequency regions, which is particularly advantageous for large systems, and in frequency regions with high densities of states, as it does not require to solve eigenvalue equations for all contributing states and individual transitions matrix elements between them.^236^ It also allows to compute nonlinear transition properties in the vicinity of additional resonances, *e.g.*, the resonant inelastic X-ray scattering (RIXS) transition amplitudes,^237^ as well as properties at imaginary frequencies, like the C_6_ dispersion coefficient.^232,238−240^

A number of implementations of damped response theory have been presented in the past two decades for time-dependent density functional theory and a variety of wavefunction methods.^80,113,232,235−238,241−254^ TURBOMOLE is, to the best of our knowledge, the only program to date that offers damped linear response functions at the RI-CC2 level of theory^232,253^ (available since release V7.7).

#### 3.9.1 Damped Response for Multiscale Modeling

DFT-based damped response implementations in TURBOMOLE cover IR spectroscopy, VCD, and Raman spectroscopy, as well as absorption and electronic circular dichroism (ECD) in the visible and ultraviolet spectral range. Furthermore, this approach has recently been extended to the modern framework of the *GW*-BSE method, being especially useful for core excitations.^113,255^

At a given complex frequency ω_ex_ = ω + *iγ*, where ω and γ are the real and imaginary parts of the external field, respectively, the coupled perturbed equation^254,256^
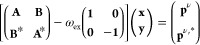
2is first solved. **A** and **B** are defined as
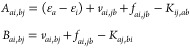
ε_*p*_ marks orbital or quasiparticle energies, *v*_*pq*,*rs*_ is a Coulomb integral, *f*_*pq*,*rs*_ is the exchange-correlation kernel, if present, and *K*_*pq*,*rs*_ is a exchange integral. The precise kinds of *f*_*pq*,*rs*_ and *K*_*pq*,*rs*_ depend on the chosen method.^254^**p**^ν^ describes the external perturbation, *e.g.*, an electric or magnetic field.^254,256^ The polarizability can then be calculated as
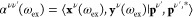
3

**Figure 11:**
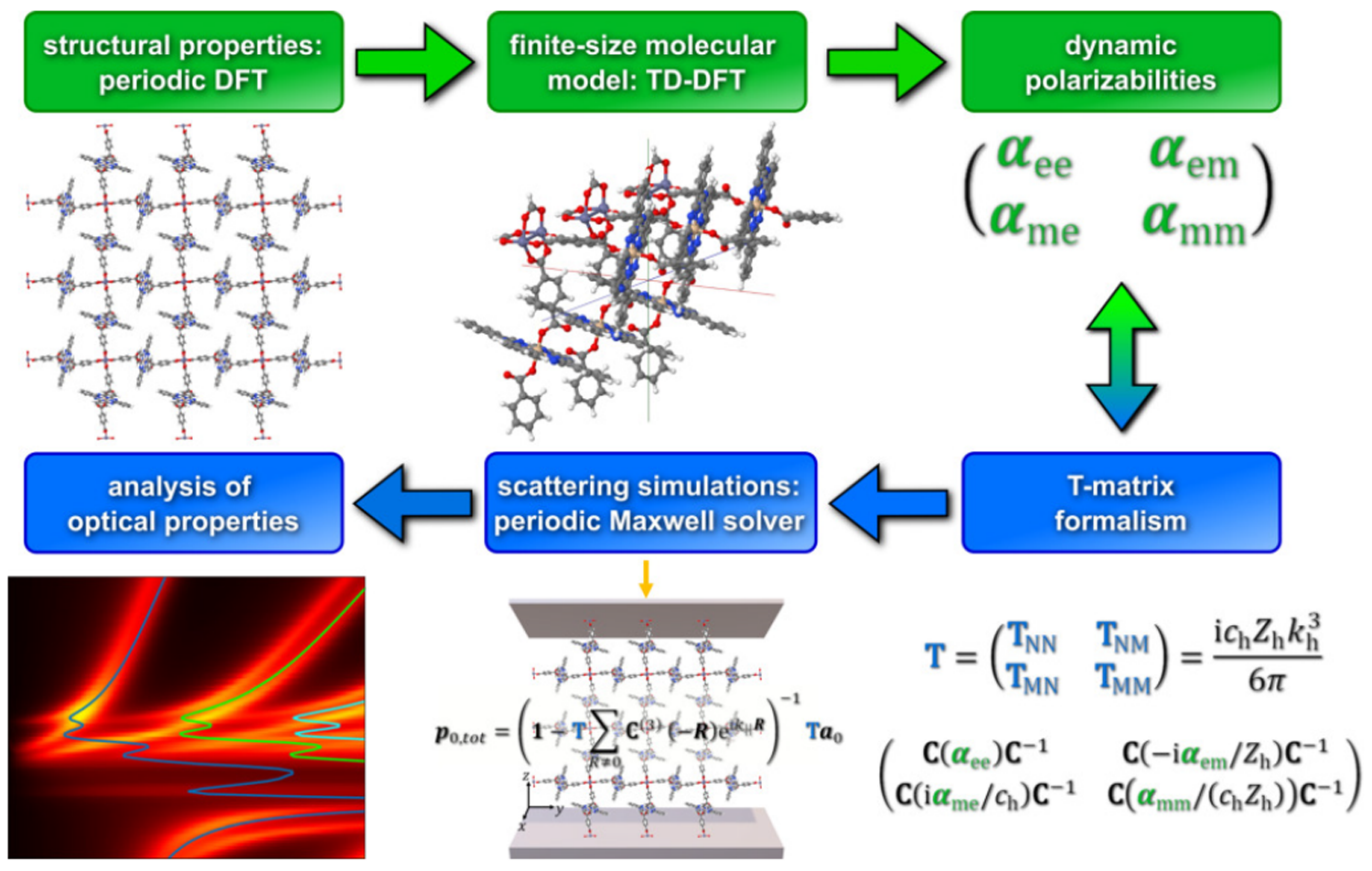
Scheme for T-matrix-based multiscale modeling of light–matter interactions using damped response polarizabilities as outlined in ref (254). Reprinted with permission from ref (254) under a CC BY-NC-ND license. Copyright 2022 the Authors.

Damped polarizabilities and the related magnetizabilities have further emerged as the quantum-chemical cornerstone of the transition matrix (T-matrix) based approach for multiscale modeling of light–matter interactions.^254,257,258^ This way, the functionalization of molecular structures within optical devices is possible. In that regard, optical cavities filled with molecular materials or metasurfaces of cylinders consisting of molecular materials can be designed. These devices exhibit tailored optical properties for a variety of applications, such as enhancing the circular dichroism of a chiral molecule. Through the multiscale approach depicted in Figure 11, the properties of a molecular unit and the macroscopic sample can be distinguished and combined to achieve a specific effect. Combining TURBOMOLE and, for example, the multilayered periodic general Mie method (mpGMM),^259^ simulations and predictions of the light–matter interactions of layer-structured materials ranging from a few to hundreds of nanometers are now routinely possible.^254,260^ To target molecular materials of arbitrary shape, the T-matrix approach was furthermore coupled with state-of-the-art homogenization techniques.^261^ Combining classical electrodynamics and quantum mechanics has proven to be a worthwhile approach in the field of light–matter interactions, and TURBOMOLE will remain at the forefront of these developments.

#### 3.9.2 Damped Response Theory for One-Photon Absorption and CD Spectra with RI-CC2

For RI-CC2, the equations for the damped response^237,262^ of the cluster amplitudes are recast in a form that only involves effective matrices in the space of single excitations, avoiding the storage of parameters for the double excitation space.
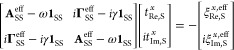
4Subscripts Re and Im represent real and imaginary components, respectively. The effective matrices in the equations above are
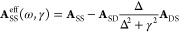
5
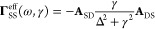
6where **A**_SS_, **A**_SD_, and **A**_DS_ are, respectively, the singles–singles, singles-doubles, and doubles-singles blocks of the CC2 Jacobi matrix. Δ^*ij*^_*ab*_ = ε_*a*_ – ε_*i*_ + ε_*b*_ – ε_*j*_ – ω are frequency-shifted orbital energy differences, and ω and γ are again the real and imaginary parts of the frequency of the external field, respectively. The effective right-hand sides are
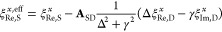
7
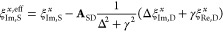
8where ξ_S_^*x*^ and ξ_D_^*x*^ are the single and doubles parts of the right-hand sides in the nonpartitioned form, respectively.^232,253^ The partitioned formulation that avoids the need to store double excitation amplitudes and four-index integrals allows applications to system sizes otherwise not accessible at the CC2 level.

**Figure 12:**
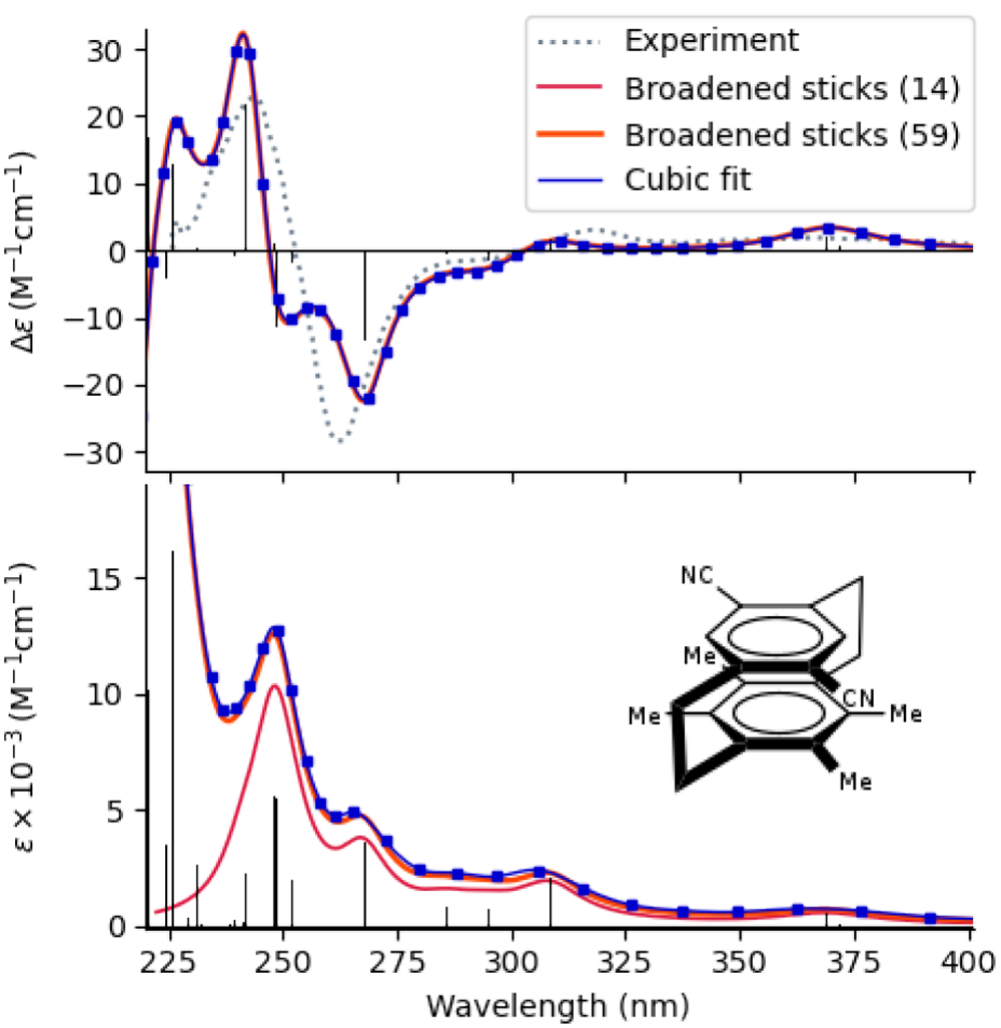
ECD (upper panel) and absorption spectra (lower panel) of (4S_*p*_)-4,7-dicyano-12,13,15,16-tetramethyl[2.2]-paracyclophane computed at the CC2/aug-cc-pVDZ level. The MP2-optimized structure and the experimental CD spectrum have been taken from ref (263). Results from damped response theory are plotted as blue squares; the blue line is a cubic spline fit to these computed points. Stick spectra are the results from state-wise calculations, and the red and orange lines are obtained by convoluting these computed stick spectra with a Lorentzian broadening with a half-width-at-half-maximum of ≈1000 cm^–1^ including, respectively, the lowest 14 and 59 states.

As an illustrative application, we computed the UV–vis absorption spectrum and electronic circular dichroism spectrum of a donor–acceptor cyclophane^263^ shown in Figure 12. The absorption spectrum was obtained from calculations of the imaginary damped dipole polarizability and the ECD spectrum from the imaginary part of the optical rotation tensor in the velocity gauge. The asymmetric form^253^ of the damped linear response function was used in the calculations (the symmetric form is also available).

Cyclophanes are well-studied examples of strained aromatic organic compounds (hydrocarbons) that exhibit planar chirality. The UV–vis spectrum is a typical example where the traditional state-wise approach converges only slowly with the number of states, as seen by the large difference between the results for 14 and 59 states that have been included for comparison. The problem does not appear in the damped response calculations.

Ongoing work is concerned with extending the implementation to induced and nonlinear spectra like MCD and RIXS.^253^

### 3.10 Nonadiabatic Molecular Dynamics Simulation for Spectroscopic Observables

Many photophysical and photochemical processes involve multiple electronic excited states coupled by radiative and nonradiative transitions. Efficient simulations of these processes by nonadiabatic molecular dynamics (NAMD) have recently become possible with transition dipole moments^30^ and nonadiabatic couplings between excited states^32,264^ computed within quadratic response TDDFT. The TURBOMOLE implementation of the multistate fewest-switches surface hopping (FSSH) algorithm enables simulations of molecular systems with 50–100 atoms and simulation times of >10 ps.^265,266^ In addition, time-resolved fluorescence (TRF) and transient absorption (TA)^34^ spectra can be simulated for comparison with experimental results.

The simultaneous treatment of multiple electronic excited states enables the examination of Kasha’s rule.^267^ According to Kasha’s rule, singlet excited states energetically located above S_1_ undergo ultrafast decay to the S_1_ state and thus are not directly involved in fluorescence or photoactivated reactions. However, exceptions to Kasha’s rule are well-known in molecules in the gas phase, such as azulene and pyrene.^268^

In our recent study, we used the NAMD implementation in TURBOMOLE to investigate the dynamics of several polycyclic aromatic hydrocarbons including pyrene, azulene, and bicyclo[6.2.0]decapentaene (BCDP, an isomer of azulene) at the PBE0/def2-SVP^269,270^ level. Azulene was found to exhibit non-Kasha behavior due to emission from the S_2_ state, in agreement with experiment.^271^ BCDP obeys Kasha’s rule and emits only from S_1_. Previous studies assigned the high-energy shoulder in the pyrene fluorescence spectrum to non-Kasha emission as a result of the reverse S_1_ → S_2_ internal conversion.^268,272−275^ Multistate NAMD simulations describe the non-Kasha behavior as a combination of S_1_ → S_2_ transitions and the change in the diabatic character of the S_1_ state from dark (L_*b*_) to bright (L_*a*_) at points of near degeneracy between the S_1_ and S_2_ states. The high-energy shoulder in the fluorescence spectrum of pyrene can be understood as originating from excited states with diabatic bright (L_*a*_) character. The S_2_ lifetime in pyrene was computed by an exponential fit of the decay of the state population as 63 fs, in agreement with the experiment value of 85 fs in methanol.^276^ The S_1_ lifetime of azulene was computed to be 2.2 ps in comparison to the experimental result of 1.4 ps in cyclohexane.^277^ The computed lifetime of the S_1_ state of BCDP was found to be 0.8 ps.

**Figure 13:**
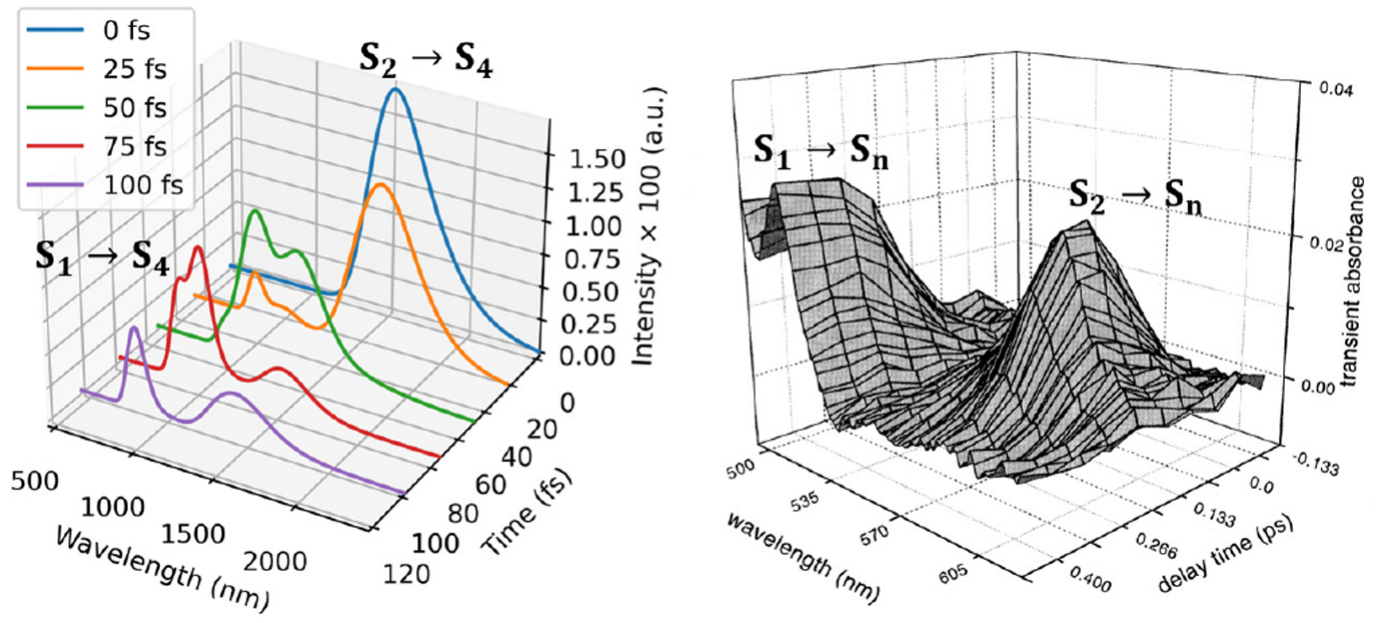
Computed (left) and experimental (right) TA spectra of pyrene in the gas phase. Band origins of S_1_ and S_2_ were shifted by −0.42 and −0.02 eV, respectively. Experimental spectra reprinted from ref (278). Copyright 1999 Hindawi Publishing Corporation. Distributed under a CC-BY license.

The NAMD trajectories were also used to obtain the TA spectrum of pyrene as an ensemble average of the Gaussian-broadened excited-state absorption and emission spectra (Figure 13). The experimental TA spectrum of pyrene shows an intense band at 580 nm and the growth of a steady state signal at around 450 nm corresponding to S_2_ → S_*n*_ transient absorption that decays rapidly and a S_1_ → S_*n*_ transition (*n* > 4), respectively.^276,278^ NAMD simulations predict S_2_ → S_4_ and S_1_ → S_4_ bands at 1500 and 800 nm, respectively. The time evolution of the S_1_ and S_2_ states is in good agreement with experiment, while the absorption maxima (λ(S_2_ → S_4_) = 1500 nm and λ(S_1_ → S_4_) = 800 nm) are red-shifted due to truncation of the electronic excitation space.

### 3.11 Generating Function Methods for Vibrationally Resolved Electronic Spectroscopy

The vibrational structure of electronic spectra gives detailed information about molecular structure^279^ and excited-state phenomena such as internal conversion and intersystem crossing.^280,281^ In the case of conformationally flexible molecules, molecular dynamics sampling often allows the shape of electronic spectra to be accounted for.^282−287^ However, this approach fails for rigid molecules with high vibrational frequencies.^288^ In this case, special care must be taken to include the quantum nature of nuclear vibrations. Efficient theoretical simulation of molecular vibrationally resolved electronic (*i.e.*, vibronic) spectra has been advanced by switching from the time-independent approach,^289^ which requires a tedious sum-overstates evaluation of Franck–Condon overlaps, to the time-dependent formalism (generating function approach), where the spectrum is given by the Fourier transform of an appropriate time-correlation function.^288,290−292^ The radless module^288,293,294^ makes use of the generating function method to compute vibrationally resolved absorption and emission spectra, as well as photoelectron ionization spectra. Spectra can be computed within the global harmonic approximation, which only requires equilibrium geometries for initial and final structures as well as vibrational spectra of both structures. The method accounts for the full Duschinsky rotation,^295^ taking into account differences in initial and final state structures and vibrational modes. Due to its efficiency, the method can be applied to large molecules, such as polyaromatic hydrocarbons.^123,124,293,296,297^ An extension of the module further allows the computation of emission and absorption spectra arising from singly occupied vibrationally excited initial states, allowing the simulation of single vibronic level (SVL) fluorescence^293^ and vibrationally promoted electronic resonance (VIPER) spectra.^298^

**Figure 14:**
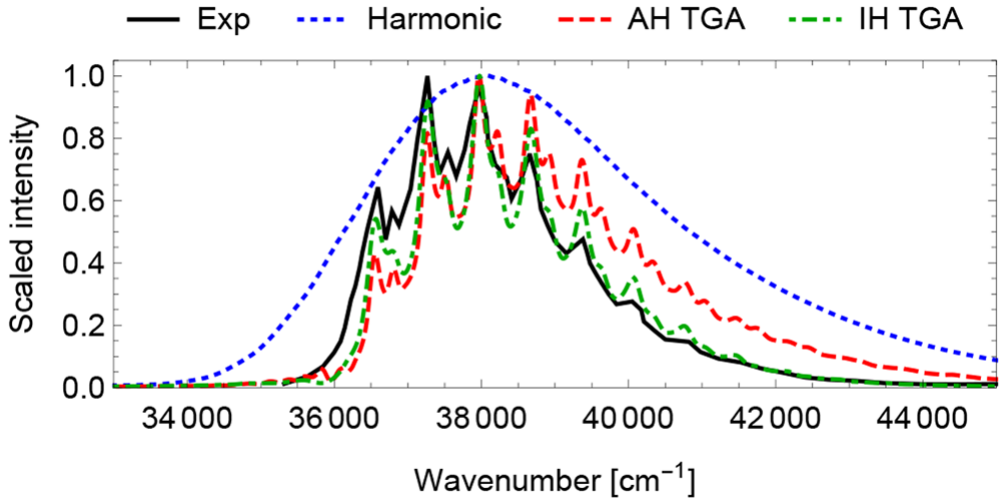
Experimental absorption spectrum of 1,2,4,5-tetrafluorobenzene (black) compared with the spectra simulated using the (adiabatic Hessian, AH) global harmonic method (“Harmonic”, blue dotted line) and single-Hessian TGA with either the adiabatic Hessian (AH, red dashed line, evaluated in the excited electronic state at its corresponding optimized geometry) or the initial Hessian (IH, green dash-dotted line, ground-state Hessian computed at the ground-state optimized geometry). All electronic structure calculations, including geometry optimization, energies, and forces for the *ab initio* dynamics and Hessians, were performed at the CC2/def2-TZVP level. Adapted with permission from ref (294). Copyright 2022 American Chemical Society.

In addition, the newly implemented semiclassical thawed Gaussian approximation (TGA)^291,299^ offers an efficient way of evaluating the time-correlation function without resorting to the global harmonic approximation. The relation between vibronic spectroscopy and semiclassical dynamics stems from the wavepacket autocorrelation picture of the dipole time correlation function, first popularized by Heller.^290^ In TGA, an initial Gaussian wave function is evolved under an effective local harmonic potential constructed at each step about the center of the wavepacket. As a result, its Gaussian form is conserved; the center of the wavepacket follows a classical trajectory, while its width is adjusted according to the instantaneous Hessian of the potential energy surface (PES). Whereas in the original *ab initio* TGA^300−303^ the Hessian of the potential energy is updated over time, in the single-Hessian version,^304−306^ implemented in TURBOMOLE as part of the radless module,^294^ the Hessian is kept constant throughout the dynamics. Therefore, the overall additional cost compared to the conventional harmonic approximation is that of a single *ab initio* trajectory in the final electronic state, which is simulated using the frog module. Since the trajectory experiences the true anharmonic PES, the method can account for anharmonicity at least approximately, although it cannot describe more subtle quantum dynamics, such as wavepacket splitting or tunneling. The TGA approach has proven to be especially useful in systems with a large displacement between the ground- and excited-state minima and in systems with a double-well-shaped PES along a low-frequency mode in the final electronic state. In such molecules, the harmonic approach typically fails because the global harmonic PES constructed around one of the wells is not adequate. Moreover, in contrast to the global harmonic methods, the TGA results often depend weakly on the choice of the Hessian, as illustrated in Figure 14. Overall, the implementation in TURBOMOLE combines these advantages of TGA with accurate and efficient excited-state electronic structures, such as ADC(2) and CC2 methods.

### 3.12 Molecular Properties from Self-Consistent GKS-spRPA

The generalized Kohn–Sham semicanonical projected random phase approximation (GKS-spRPA) provides a route for obtaining one-particle energies at the RPA level of theory.^16^ These one-particle energies provide accurate estimates of ionization potentials (IPs) and electron affinities due to a correct description of orbital correlation and relaxation effects. Its computational cost was reduced from  to  using well-known analytic continuation (AC) techniques.^307−309^

#### 3.12.1 Applications to Nonvalence Anionic States and X-ray Emission Spectroscopy

The AC version of GKS-spRPA retains a high accuracy across energy scales from valence to core-ionization energies. The versatility of the AC GKS-spRPA was shown by its application to problems involving very weakly bound anionic states and very strongly bound core-hole ionization energies.

**Figure 15:**
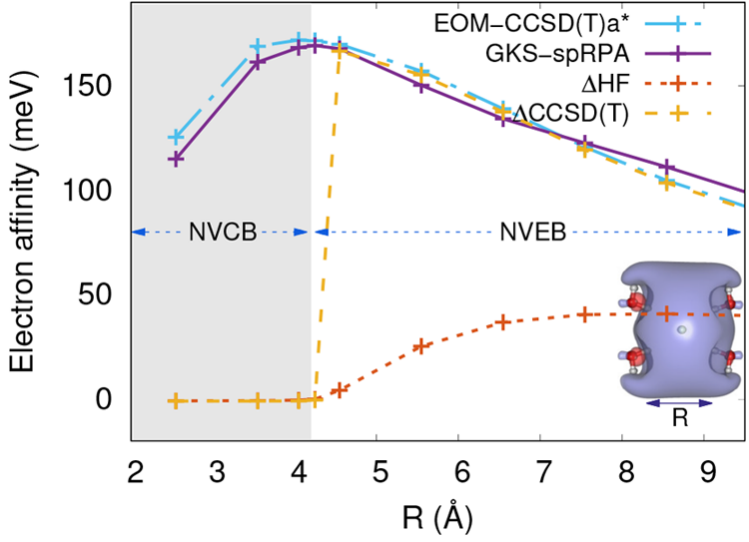
EAs of a water tetramer cluster for different dimer separations (*R*) computed using GKS-spRPA, Δ*H*F, ΔCCSD(T), and EOM-CCSD(T)a* methods. The inset shows the arrangement of the tetramer with a 70% isosurface of the LUMO at *R* = 4.047 Å. aug-cc-pVDZ basis sets were used for O and H atoms, and a 7*s*7*p* set of basis functions located at the center of the cluster was used. The shaded (unshaded) area of the plot corresponds to NVCB (NVEB) anionic states. Reprinted with permission from ref (309). Copyright 2021 American Chemical Society.

Nonvalence anionic states of molecules are weakly bound states where the excess electron occupies a diffuse orbital. The excess electron is bound by a combination of long-range electrostatic and correlation effects. When electrostatics are sufficient to bind an excess electron, the anionic states are referred to as nonvalence electrostatic-bound (NVEB), and when correlation effects are necessary the states are referred to as nonvalence correlation-bound (NVCB). Using a model water tetramer cluster, the GKS-spRPA approach was shown to provide electron affinities (EAs) from the lowest unoccupied molecular orbital (LUMO) energies that were within 10 meV of those provided by the EOM-CCSD(T)a* approach, see Figure 15. The high accuracy of the GKS-spRPA approach is due to the correct description of long-range correlation effects. The AC approach introduces errors of less than 5 meV for nonvalence states.

**Figure 16:**
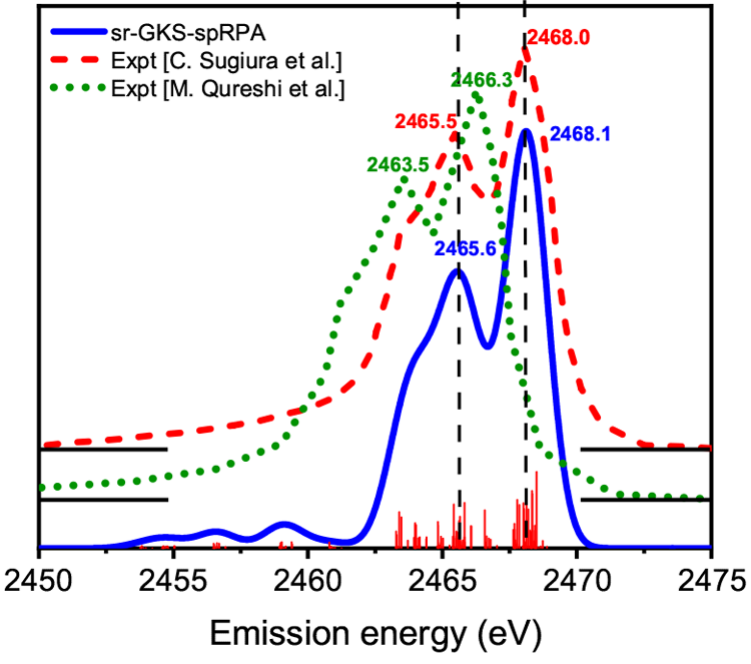
XE spectra of S_8_ from experiments^311,312^ and the SR-GKS-spRPA method.^310^ The computed spectrum (in blue) was obtained by Gaussian broadening of the vertical transitions (red lines) using a width parameter of 1 eV. The vertical dashed lines denote the two intense peak positions for SR-GKS-spRPA. Reprinted and adapted with permission from ref (310). Copyright 2022 American Chemical Society.

The AC GKS-spRPA method can also be used for simulating nonresonant X-ray emission (XE) spectra using just the information from the one-particle eigenspectrum.^310^ XE energies, *ΔE*, computed by taking the difference between core and valence IPs and the oscillator strengths, *f*_osc._, are evaluated within a frozen orbital approximation
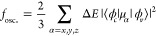
9where ϕ_*c*_ and ϕ_*v*_ are the core orbital and valence GKS-spRPA orbitals involved in the XE process, respectively. The AC GKS-spRPA approach was used in conjunction with the scalar-relativistic (SR) X2C approach and uncontracted basis sets to estimate highly accurate XE spectra for molecules containing second- and third-period elements, for example, see Figure 16. Using uncontracted basis sets, the XE energies were found to have MAEs of 0.7 eV for both second and third period-based XE. The X2C-based AC GKS-spRPA approach thus enables the simulation of nonresonant X-ray emission within a simple one-particle picture while avoiding the use of empirical shifts or core-hole reference states. The latter is an appealing aspect, since issues related to variational instability, which are present in core–hole reference based methods, are avoided.

#### 3.12.2 Orbital Ordering in Quinacridone

**Figure 17:**
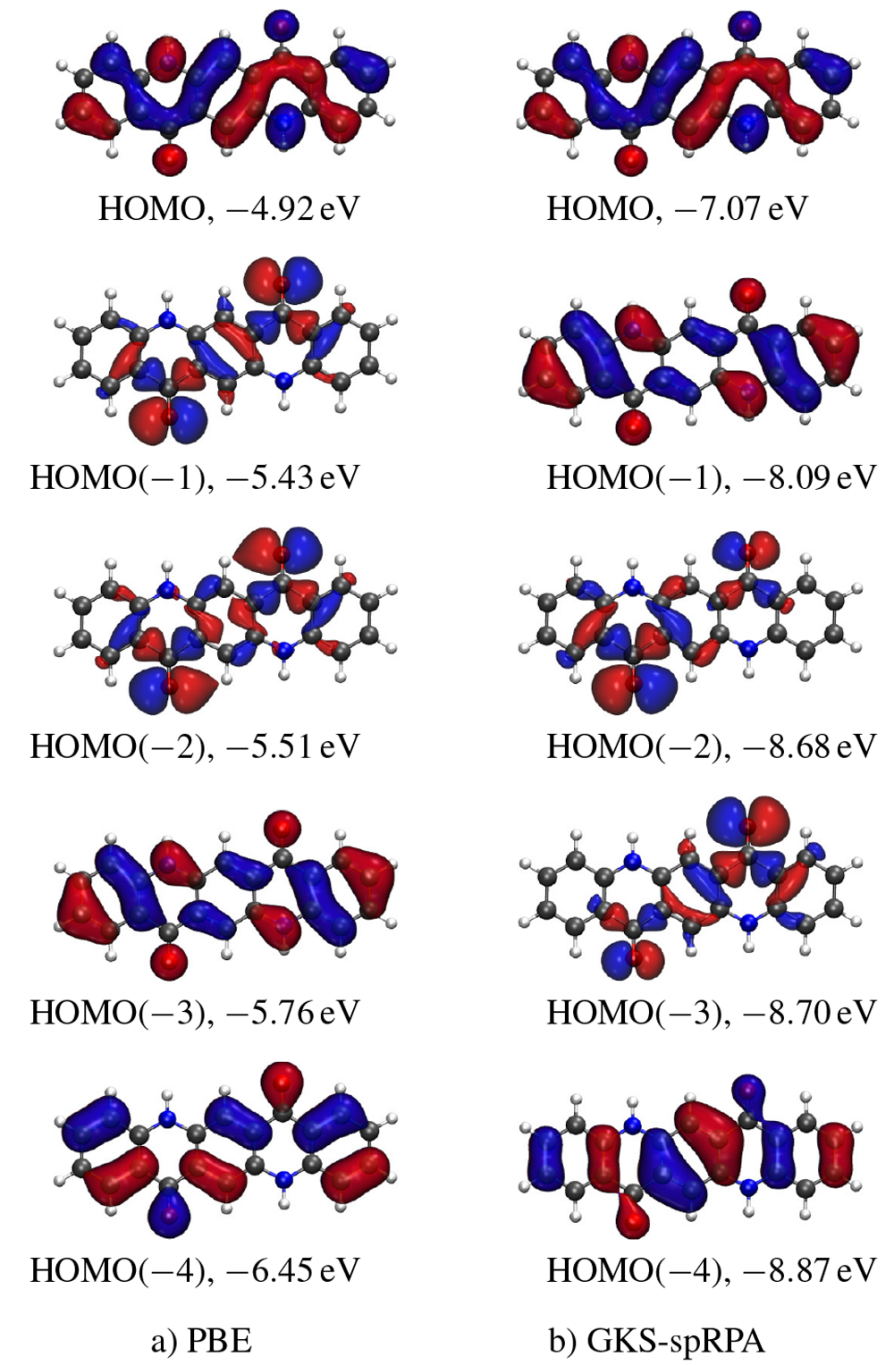
Plots of the five highest occupied orbitals at isovalues of ±0.02 shown for (a) PBE and (b) GKS-spRPA (with the PBE potential). The structure of quinacridone was optimized with PBE-D3^313−315^ and cc-pVTZ basis sets.^316^ Reproduced from ref (317) with the permission of AIP Publishing.

The accuracy of GKS-spRPA^16^ for ionization potentials (IPs) compared to nonselfconsistent RPA^23^ is due to partial satisfaction of functional self-consistency, which requires that the Kohn–Sham (KS) density equal the interacting density defined as the functional derivative of the ground-state energy with respect to the external potential.^16,317^ A comparison of the GKS-spRPA orbitals and orbital energies to experimental photoelectron spectra of quinacridone illustrates this point, see Figure 17. Within both the KS and the GKS approach, the HOMO energy equals the first IP and subsequent lower-lying orbital energies approximate higher principal IPs.^318,319^ However, this is often not true for semilocal density functional approximations. PBE^313^ predicts a HOMO(−1), HOMO(−2), and HOMO(−3) ordering inconsistent with the results of optimally tuned range-separated hybrid functional (OT-RSH) calculations, yielding an accurate description of experimental photoelectron spectra and *G*_0_*W*_0_ IPs.^320,321^ The canonical GKS-spRPA orbital ordering qualitatively agrees with the one OT-RSH one down to HOMO(−3), see Figure 17. Furthermore, the negative GKS-spRPA HOMO energy of 7.07 eV is close to the experimental IP of 7.23 eV,^322^ whereas the negative PBE HOMO energy of 4.92 eV is significantly too small.

### 3.13 Subquadratic-Scaling PNO-CCSD(T) and PNO-CCSD(T)-F12 Methods

Since 2010, TURBOMOLE has supported accurate wave function methods for computing the ground-state energies of molecular configurations, and up to CCSD(T) and BCCD(T) are available, together with their explicitly correlated counterparts CCSD(T)(F12*) and BCCD(T)(F12*).^68,69^ When combined with a sufficiently large basis set, these methods deliver ground-state energy differences for reaction or interaction energies and potential energy surfaces to an accuracy that enables a quantative comparison with experimental results. The characteristics of the TURBOMOLE implementation are low memory and disk requirements and shared-memory parallelization, which make calculations on systems with ∼20 atoms routinely possible on modern machines.

To evaluate energies for larger molecules, the steep  scaling of the costs with system size must be overcome. Local approximations based on the short-ranged nature of electron correlation in molecules provide a route to near-linear scaling of costs with the system size. The PNO approach uses an approximate MP2 pair density to construct the set of local virtual orbitals for each pair that is best suited to describing the correlation of that pair. This, in combination with screening and local density fitting approximations, among others, reduces the scaling from  to  in the asymptotic limit. For ground-state energies, PNO-MP2, PNO-CCSD, and PNO-CCSD(T) explicitly correlated variants PNO-MP2-F12, PNO-CCSD(F12*), and PNO-CCSD(T)(F12*) have been available since V7.6.^70−73^

The efficiency of the implementation is greatly improved if the PNOs are expanded in projected atomic orbitals (PAOs) rather than directly in terms of atomic orbitals (AOs). This is known as domain-based local PNO theory (DLPNO). In TURBOMOLE, the PAO domains for each pair are determined also using the approximate MP2 density rather than by analyzing the MO coefficients and are consequently much more compact. The resulting domains are called principle domains.^323^ Very recently, the principle domain approach has been extended to F12 theory, where principle domains and PNOs are required for every subspace in the F12 strong orthogonality projector.^324^ In contrast to implementations in other software packages, we do not use the simplified “A” approximation^95^ for the MP2-F12 contributions. This has the benefit that the energies converging smoothly to both the canonical limit and the basis set limit can be extrapolated using PNO extrapolation techniques.^325^

**Figure 18:**
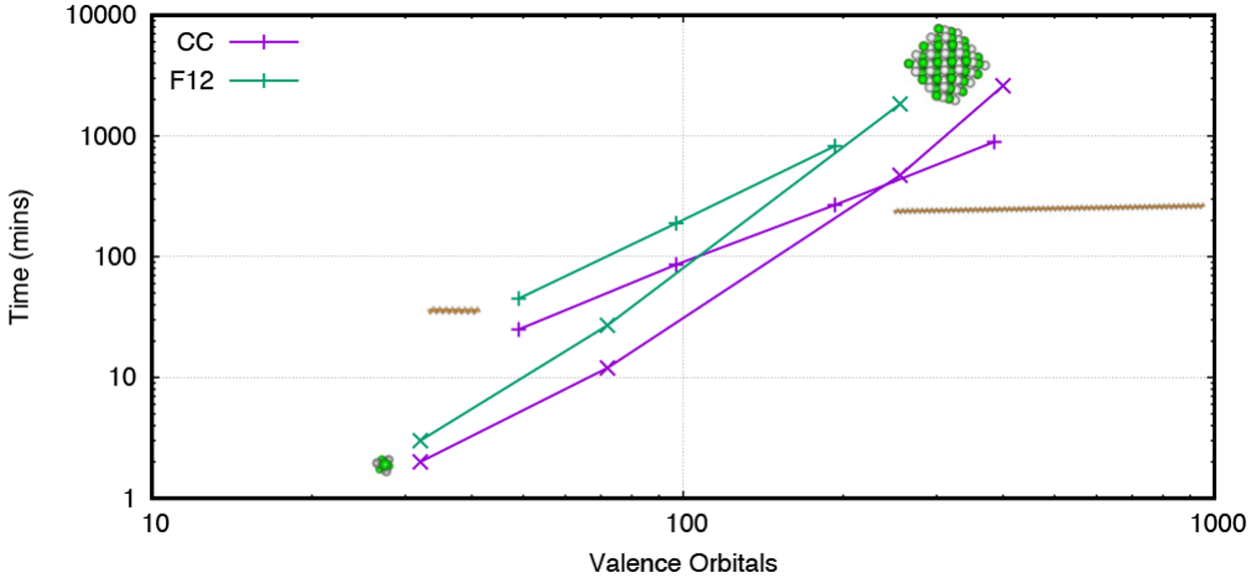
Wall-clock timings of PNO-CCSD(T) and PNO-CCSD(T)(F12*) calculations for alkane chains (+) and NaCl clusters (cross).

To illustrate the characteristics of the TURBOMOLE implementation and what is now possible, we report timings of PNO-CCSD(T) and PNO-CCSD(T)(F12*) calculations on a sequence of alkane chains and rock salt crystal fragments in Figure 18. The default tight (10^–7^) PNO truncation threshold was used in all cases. The def2-TZVPP basis and def2-TZVP were used for C_*n*_H_2*n*+2_ and Na_*n*_Cl_*n*_, respectively, and calculations were run on a 48 core Intel processor with 200 Gb of memory. For the linear systems, the observed scaling is subquadratic, with C_128_H_256_ taking 15 h to complete. For the globular systems, the observed scaling is subcubic, with Na_50_Cl_50_ taking 45 h to complete. The F12 calculations are 2–3× more costly than non-F12 calculations but provide energies close to the basis set limit without requiring basis sets with a large number of AOs per atom.

### 3.14 Real-Time TDDFT for Molecules

While linear-response TDDFT can be used to study excitations under weak fields, a better understanding of nonlinear excited state dynamics at the femto- and sub-femtosecond time scales requires using RT-TDDFT.

**Figure 19:**
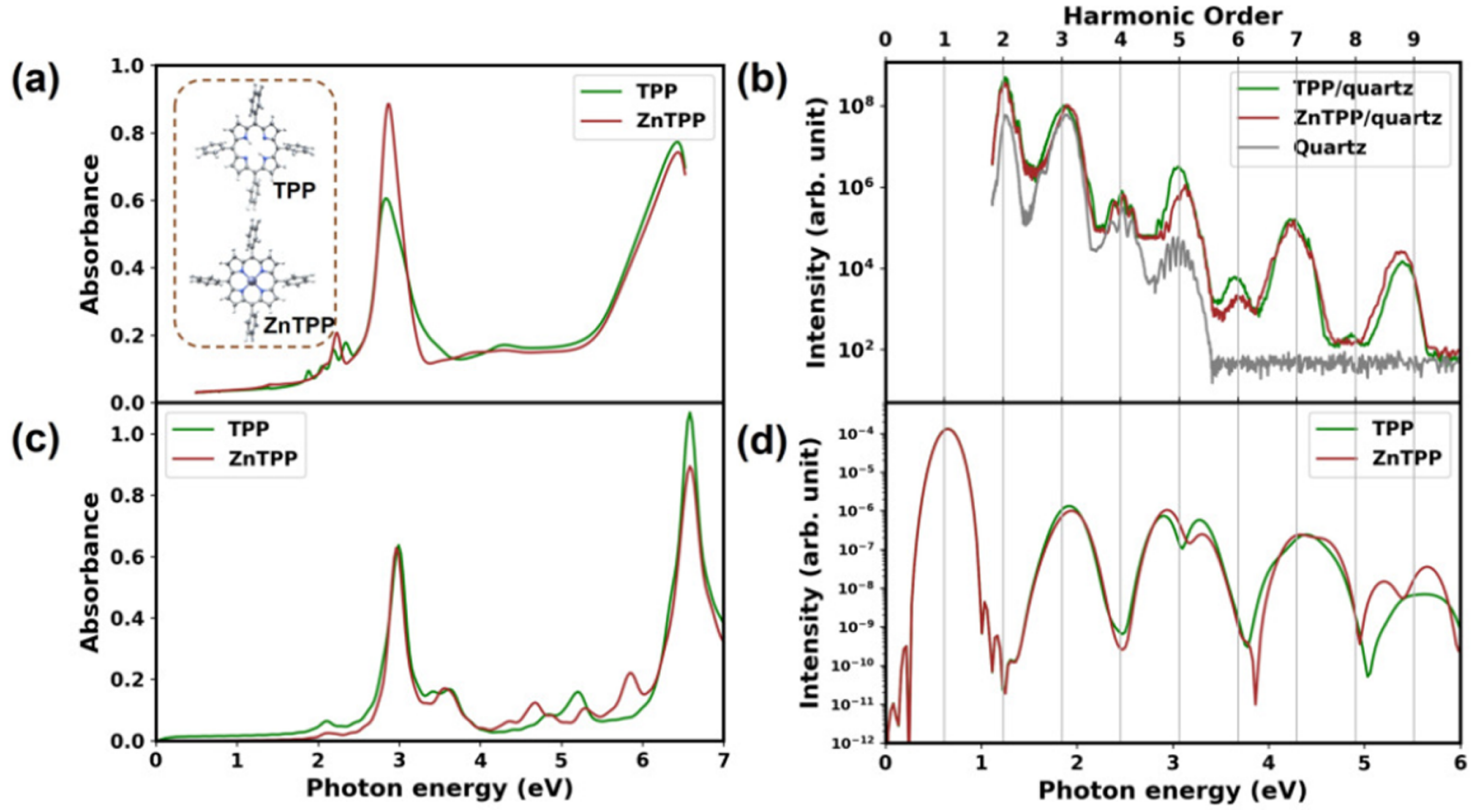
(a) Experimental linear absorption spectra and (b) HHG spectra of 100 nm thick TPP and ZnTPP samples. (c) Calculated linear absorption spectra and (d) HHG spectra of TPP and ZnTPP molecules.^326^

RT-TDDFT for molecular systems has been implemented^89^ within the riper module^55−58,327,328^ using the Magnus propagator^329^ and the predictor-corrector scheme^330^ for time integration. The implementation utilizes density fitting and continuous fast multipole method (DF-CFMM) techniques^56^ to speed up the KS matrix evaluation and scales almost quadratically with system size. Previously, the implementation was used to simulate absorption spectra over a wide range of frequencies by perturbing the molecules with weak electric fields.^89^ Recently, the code has been extended to simulate high harmonic generation (HHG) spectra under intense laser pulses and was utilized to complement and analyze the pulse-induced electron dynamics in the organic semiconductor molecules tetraphenylporphyrin (TPP) and zinc-tetraphenylporphyrin (ZnTPP).^326^Figure 19 shows the good agreement of the experimental absorption and HHG spectra with the calculated ones. The difference in the first few harmonics is due to the fact that the experimental spectra contain contributions from the quartz substrate. Overall, the HHG and absorption spectra of both the porphyrins are quite similar due to the very similar electronic structure. Our simulations combined with experiments showed that π → π* excitation plays a major role in the harmonic generation process in porphyrins. It was also discovered that resonant excitation leads to an early onset of nonperturbative behavior for the fifth harmonic, and similar effects are expected in Brunel harmonic generation with other organic materials.^326^

### 3.15 Developments of the DFT-Based Embedding Implementations

#### 3.15.1 Frozen Density Embedding Implementation

Environment effects on molecular systems beyond static Coulomb potentials can be treated in TURBOMOLE with the conductor-like screening model (COSMO), polarizable embedding (PE), or frozen density embedding (FDE). Developments on COSMO and PE in TURBOMOLE have been presented in the previous review.^92^ In contrast to the latter approaches, FDE is an atomistic QM/QM embedding model, which compared to QM/MM schemes uses a purely quantum-mechanical description of the total system and does not require any system-specific parametrizations prior to a calculation.

The implementation of FDE in TURBOMOLE was until recently restricted to just two subsystems, limiting its applicability. It has now been extended to handle arbitrarily many subsystems and to an FDE variant free of intermediates evaluated on the supermolecular orbital basis. For the embedding potentials, the newly implemented approach uses only electron densities and electrostatic potentials of the subsystems, which are computed on an integration grid generated for the total system. It is available for HF and DFT within the dscf and ridft programs. In combination with the ricc2 program, it can be used to compute ground-state energies with MP2 and CC2 within the perturbation to the energy (PTE) or post-SCF reaction field coupling approaches. Excitation energies can be calculated within the frozen solvent approximations either with or without the kernel contributions. An important feature of the new implementation is the possibility to include pseudopotentials to improve the embedding potential. This allows for a more accurate description of the Pauli repulsion,^83^ which is particularly important for electronically excited states to prevent unphysical charge spill-out. Besides all of the new features in FDE, it should also be mentioned that the current implementation is still limited to closed-shell subsystems.

**Figure 20:**
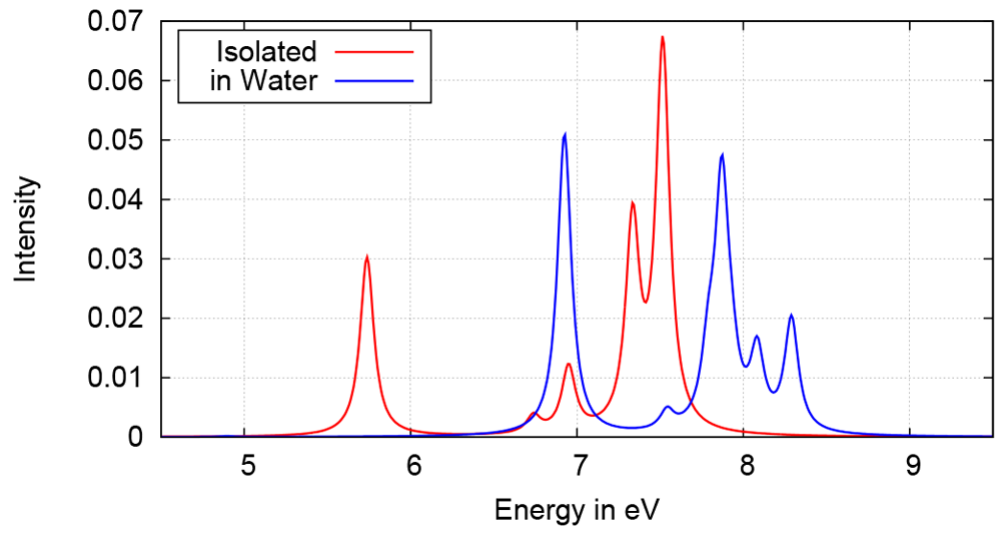
Computed spectra of acetone isolated (red) and surrounded by 237 water molecules (blue).

To demonstrate its capabilities, we computed the first eight excitation energies of acetone at the CC2/aug-cc-pVDZ level embedded in 237 water molecules within the frozen solvent approximation. The active subsystem also includes two water molecules beside acetone and was treated with the HF method during the freeze and thaw (FaT) cycles, while the remaining water molecules were treated as separate subsystems using DFT with the PBE exchange correlation functional^313^ and a gridsize of 3.^9,331^ For the embedding potential, the PBE exchange correlation and the LC94 kinetic energy functional^332^ with a gridsize of 3 were used. A comparison of the spectrum of solvated acetone with that of isolated acetone is shown in Figure 20.

Recently, another update scheme of the subsystem densities was implemented, where the densities of all subsystems are updated simultaneously at the end of a FaT cycle instead of successive updates immediately after each subsystem calculation. Thus, the updated densities of each subsystem only contribute to the embedding potentials in the next FaT cycle, offering the advantage that interim results no longer depend on the enumeration of the subsystems; this allows the subsystem calculations to be performed simultaneously to improve efficiency in parallel runs. In Table 3, the wall times for both update schemes are compared for an reduced example of the above system containing only 78 water molecules. The timings show that this kind of parallelization leads to a decrease of the wall time in the case of many small subsystems where it is inefficient to parallelize single subsystem calculations over many CPU cores.

**Table 3:** Wall Times for an Acetone Molecule in a Water Cluster for the Two Different Update Schemes^a^

update scheme	successive	simultaneous
*T*(40), min	122.8	71.4
*T*(1), min	369.6	586.2
speedup	3.0	8.2
*n*_FaT_	6	10

a *T*(*X*) represents the computational wall time obtained using *X* CPU cores. *n*_FaT_ gives the number of FaT iterations where it is necessary to reach convergence.

Future developments on the implementation of the FDE method aim to increase the applicability to systems containing large subsystems by combining it with PNO-based methods^333^ and to reduce further the computation time of the Coulomb contribution to the embedding potential.

#### 3.15.2 FDE and Projection-Based Embedding for Molecules and Solids

The FDE implementation described above is suitable for molecular and weakly interacting subsystems, as it employs embedding potentials based on approximate kinetic energy density functionals (KEDFs).^334^ Recently, a DFT-based embedding scheme that treats both molecular and periodic systems on equal footing has been implemented within the riper module.^82^ This implementation supports both FDE and projection-based embedding^335^ (PbE) via a level-shift projection operator^336^ (LSPO). PbE along with FaT cycles can be used to perform exact DFT-in-DFT embedding for molecules and solids and reproduce the exact total DFT energies, even for strongly interacting subsystems. Similar to the implementation in section 3.15.1, the embedding scheme is also coupled with correlated wave function (CW) methods and additionally with RT-TDDFT, enabling CW-in-DFT and RT-TDDFT-in-DFT calculations, respectively, on a cluster embedded in a molecular/periodic environment. However, here the CW-in-DFT calculations are performed only by adding the DFT-based embedding potential as a static term to the HF core potential of the active subsystem and obtaining the converged HF reference orbitals for post-SCF calculations. RT-TDDFT-in-DFT, on the other hand, does support updating a portion of the embedding potential during the time evolution in select cases.

**Figure 21:**
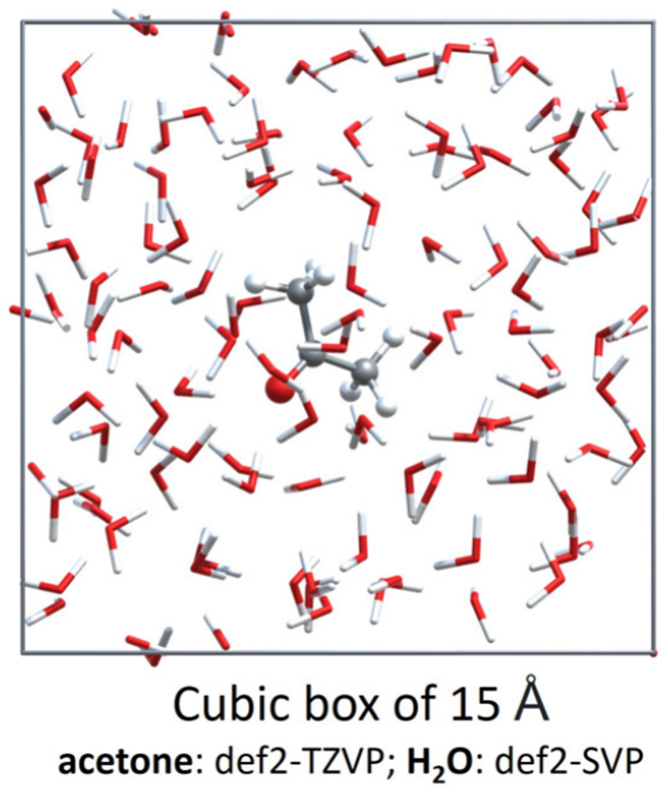
Acetone + H_2_O in a cubic box of 15 Å containing 113 water molecules visualized using CrysX-3D Viewer.^337^

As an illustrative application of the work, Figure 21 and Table 4 show that the solvatochromic shift in the first excitation energy, calculated using the CC2-in-DFT method (molecule-in-periodic) for an acetone molecule (active) solvated with 113 water molecules (periodic environment) in a cubic box with 3D periodicity, is in remarkable agreement with the shifts calculated using standard CC2^46^ for acetone + (H_2_O)_20_, acetone + (H_2_O)_35_, and acetone + (H_2_O)_48_ clusters at only a fraction of the computational cost. While beneficial, it is also important to acknowledge the limitations of the implementation as well, such as being restricted to only two closed-shell subsystems and using only LDA/GGA functionals for the embedding potential.

**Table 4:** Solvatochromic Shift *ΔE* of Acetone (Ac) S_1_ Excitation in Water Calculated via CC2 on Ac + (H_2_O)_*n*_ Clusters and CC2-in-DFT (Periodic)^a^

	CC2			CC2-in-DFT (periodic)
	Ac + (H_2_O)_20_	Ac + (H_2_O)_35_	Ac + (H_2_O)_48_	Ac in 3D H_2_O
*ΔE*, eV	0.19	0.19	0.21	0.20
*N*_bf_	640	1000	1312	2872
*T*, h	0.88	8.36	37.33	4.10

a *T* denotes the wall time of the CC2 solvatochromic shift calculations.

### 3.16 Periodic Hartree–Fock Exchange

**Figure 22:**
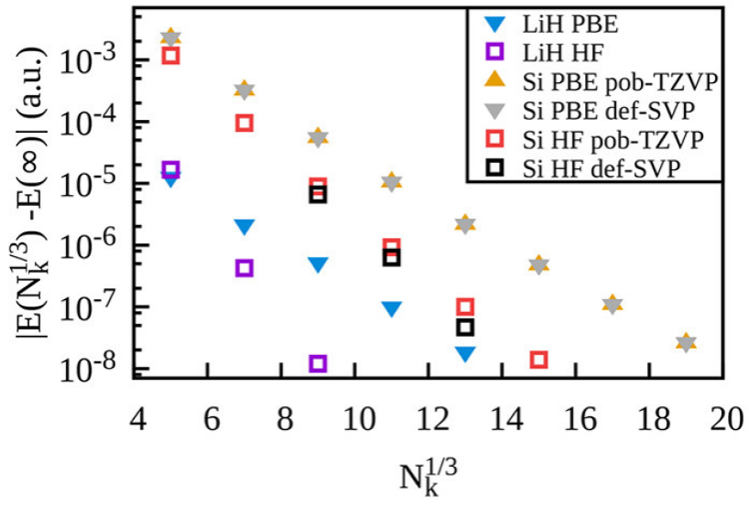
Self-consistent total energy differences |*E*(*N*_**k**_^1/3^) – *E*(∞)| per primitive cell for the PBE and HF methods. LiH is calculated in the rocksalt structure with a lattice constant of 4.084 Å^339^ and is described with the basis set from ref (340). For Si, we use the diamond structure with a lattice constant of 5.430 Å^341^ and the pob-TZVP^342^ and def-SVP^343^ basis sets. *E*(∞) is approximated by the energy obtained with a 31 × 31 × 31 *k*-mesh, since |*E*(25) – *E*(31)| < 4 × 10^–10^ a.u. for LiH and Si. Reprinted with permission from ref (328). Copyright 2018 American Chemical Society.

In ref (328), we have presented a robust implementation of the periodic Hartree–Fock exchange in TURBOMOLE’s riper module.^56,57^ Without precautions, exchange matrix elements may be divergent, arising from an artificial periodicity of the density matrix. This periodicity of the density matrix is introduced in practical calculations by the discretization of wavevectors. The finite *k*-mesh determines in turn the size of the Born–von Kármán supercell. We have demonstrated^328^ that a minimum image convention^338^ removes the divergence for discrete *k*-meshes. While calculations with periodic HF exchange may be unstable for small supercells, stable SCF calculations and convergence of total energies are typically achieved for sufficiently large sizes of the supercells. The size of the supercell or *k*-mesh that is required for a reliable energy depends on the locality of the density matrix and hence both the electronic structure of the studied material and the chosen basis set. For selected insulators and semiconductors, we have demonstrated that HF total energies converge exponentially with the number of *k*-points,^328^ see Figure 22.

Through our implementation of periodic exchange, conventional Hartree–Fock calculations can be carried out with TURBOMOLE for periodic systems of any dimension. In addition, DFT calculations with hybrid functionals can now be performed routinely for semiconductors and insulators, and we showed successful applications of PBE0^313,344^ and HSE06^345^ hybrid and range-separated hybrid functionals.^328^ As the next important step, analytical gradients shall be added for structure optimization. Furthermore, the Hartree–Fock exchange infrastructure that is available may be used in the development of new electronic structure methods for periodic systems that require exchange.

## 4 Select Features under Development

### 4.1 Nuclear Electronic Orbital Method

Proton-coupled electron transfer (PCET) reactions are an important class of reactions that cannot be adequately described within the Born–Oppenheimer approximation.^346^ A remedy to this problem is the nuclear electronic orbital (NEO) methods, which treat not only electrons but also the protons of selected hydrogen atoms quantum mechanically.^347,348^ This is of particular importance for reactions that include proton transfer, such as, for example, acid–base reactions. The corresponding effects become especially important when the proton transfer is coupled to the electronic structure, such as in photoacids and photobases. In an initial proof-of-principle implementation, nuclear electronic orbitals were made available for the Hartree–Fock method (NEO-HF) and second-order Møller–Plesset perturbation theory (NEO-MP2) in a development version of TURBOMOLE. Furthermore, for the NEO-HF method, analytical gradients have since been implemented to allow for structure and basis set optimization.

**Figure 23:**
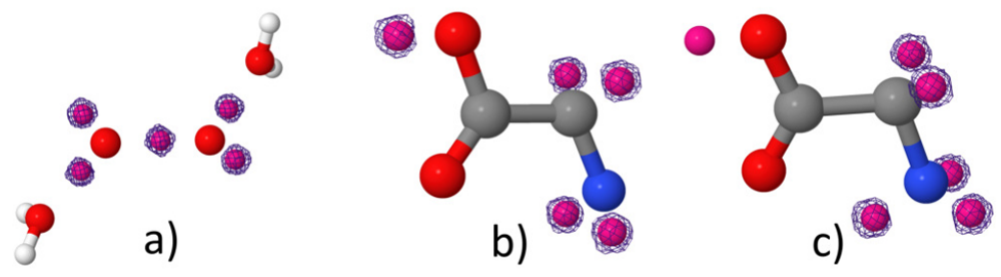
Nuclear orbitals as isosurfaces of the total densities with a cutoff of 0.0001 calculated with NEO-HF in the dscf program using the def2-TZVP basis for electrons and DZSPDN^348^ for protons. White, classical proton; pink, center for basis set of a quantum proton; red, oxygen; and blue, nitrogen. (a) *trans*-Zundel isomer of H_9_O_4_^+^, (b) neutral glycine, and (c) zwitterionic glycine.

Figure 23a shows the nuclear orbitals of the *trans*-Zundel isomer H_9_O_4_^+^.^349^ The nuclear orbital energies, as calculated by the NEO-HF method, give an estimate of the binding energies of the protons. While the four outer ones have energies from −450 to −436 kcal/mol, the central one is the least stable with an energy of −417 kcal/mol. If the outer two water molecules are removed, the energies change to between −400.5 and −399.6 kcal/mol for the four outer protons and −371 kcal/mol for the central one. This hints at the ionic cluster being stabilized by the outer water molecules.

The protons of the neutral glycine molecule in Figure 23b have orbital energies between −633 and −546 kcal/mol, with the least stable one being the one at the carboxyl group. The zwitterionic glycine structure in Figure 23c has been optimized as a positively charged system with six classical protons. A NEO-HF calculation with five protons resulted in proton orbital energies between −663 and −558 kcal/mol. This demonstrates that protonation and deprotonation at certain sites can be elegantly investigated by the *ab initio* occupation of nuclear orbitals at the respective sites instead of placing classical nuclei according to intuition.

Implementing these methods in TURBOMOLE allows for the use of highly efficient schemes that already exist for purely electronic methods. The NEO methods can also use various existing programs to analyze the results. In future developments, NEO–DFT^350^ will be implemented for molecular and periodic^351^ systems.

### 4.2 Hartree–Fock-Based Adiabatic Connection Models

MP2^352^ is one of the most used approaches for wavefunction-based correlation energies, as well as being used in the double-hybrid (DH) DFT.^353,354^ Nevertheless, the MP2 method shows several limitations, *e.g.*, it overestimates the correlation energy in large systems^355^ and diverges for systems with a vanishing gap.^356^ For this reason, several regularized and/or scaled MP2 methods have been developed.^356−358^

Another more recent path in this direction is the Møller–Plesset or Hartree–Fock adiabatic connection (HFAC) method.^359^ In the HFAC approach, the correlation energy *E*_c_ is given as a nonlinear function of *E*_MP2_, *E*_x_^HF^ (the HF exchange energy), and two semilocal functionals of the HF density (*W*_c_ = *W*_c_[ρ^HF^] and *W*_c_^′^ = *W*_c_^′^[ρ^HF^]). The latter are derived from the strong-correlation regime,^360,361^ and we have
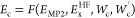
10The HFAC method *de facto* includes an infinite-order resummation of the MP correlation series thanks to the interpolation with the strong-correlation limit, as in the more conventional AC based on DFT.^362^ The nonlinear function *F* can be approximated by modeling the HFAC curve at various coupling strengths^359^ using known exact asymptotic conditions.^361,363^ Consequently, *F* satisfies two important limits:
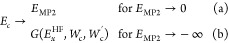
11where *G* is a nonlinear function whose form depends on the choice of *F*. For well-behaving approximations of *F*, the condition in eq 11b yields a finite energy whenever *E*_MP2_ → – ∞, thus removing one main limitation of MP2 and DH functionals for systems with a vanishing gap. The condition (eq 11a) is an exact condition,^359^ which is violated in all the regularized MP2 methods.^356,357^ Thus, the HFAC method allows us to overcome the main drawbacks of the MP2 approach within a well-defined theoretical framework at the small extra cost of a post-HF semilocal DFT calculation.

Some working approximations of *F* have been proposed (*e.g.*, ISI,^362,364^ RevISI,^365^ and MPACF1^363^), and they have been implemented in TURBOMOLE together with the currently available DFT approximations for *W*_c_ and *W*_c_^′^.^360,366^ Note that eq 10 is not size-consistent for systems composed of different species of fragments (as *F* is a nonlinear function). However, a size-consistent correction (SCC)^367^ can be readily computed with TURBOMOLE at no additional costs, allowing the calculation of dissociation curves.

**Figure 24:**
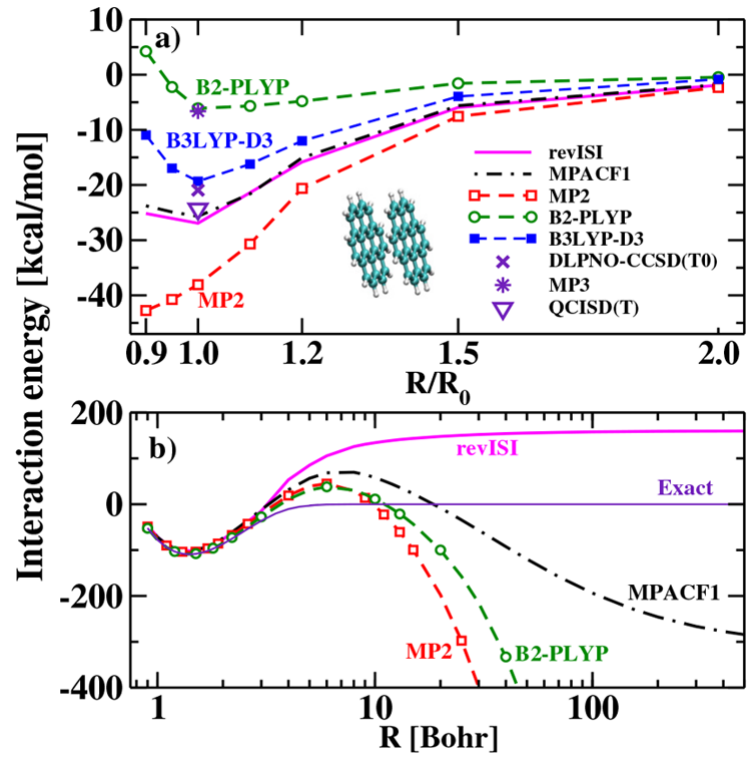
Dissociation energy curves for (a) a coronene dimer and (b) H_2_, as computed with different methods. For the coronene dimer, reference data are taken from the literature,^368,369^ and the equilibrium distance is *R*_0_ = 3.458 Å. All calculations were performed with the *hfacm* script. The coronene dimer results are based on a complete basis set extrapolation from cc-pVQZ results. H_2_ calculations employed the aug-cc-pVQZ basis set. The strong-correlation functional used in the HFAC results is the hPC model.^366^

Two examples of applications where MP2 and DH functionals fail whereas the HFAC implementation can be readily used are displayed in Figure 24.

Figure 24a shows the dissociation curve for the coronene dimer, a prototype for a wide class of problems that are hardly tractable with high-level correlated wave function methods but are poorly described by MP2 because of the lack of high-order correlation contributions.^355^ In constrast, HFACM methods are very close to the available reference data at the equilibrium geometry. The MPACF1 functional has been tuned on dispersion complexes and on average exhibits an error 2–5× smaller than MP2.^363^

Figure 24b shows the H_2_ dissociation in a restricted formalism, a prototype of a strongly correlated system.^129−131,357,364^ All methods work well close to the equilibrium geometry. However, for a larger separation where the energy-gap closes, MP2 and DH functionals rapidly diverge. HFAC methods, however, remain well behaved, yielding a finite interaction energy, see eq 11b. The exact result is not reproduced, as the available HFAC functionals are approximated and do not take into account the recent theory developments.^361^ Further development and testing are thus required, and the HFAC implementation in TURBOMOLE represents an efficient platform to this end.

### 4.3 Approximate TDDFT Approaches

Time-dependent density functional theory is still the most-used approach for the calculation of excitation energies of molecular and extended systems, thanks to its favorable accuracy/computational-cost ratio. Nevertheless, the computational cost of first-principles TDDFT calculations limits routine calculations to systems with a few hundreds of atoms, depending on the choice of the exchange-correlation (XC) functional. Different methods and algorithms have been developed in order to increase the TDDFT efficiency,^370−373^ most prominently the RI-*J* technique.^374−377^ Another efficient, though approximated, path is to perform a semiempirical tight-binding linear-response (TBLR) approximation,^378−380^ using first-principles KS orbitals and eigenvalues. TBLR methods speed up the TDDFT calculation by about two orders of magnitude. TBLR is accompanied by a loss in accuracy of about 0.1–0.2 eV, which is comparable to the overall TDDFT accuracy.^381,382^ More recently, it has been shown that, for semilocal XC functionals, the TBLR approaches can be considered as an approximation of the RI-TDDFT scheme with only one s-type Gaussian basis function per atom in the RI auxiliary basis set (TDDFT-as)^383^ and with the three index RI integrals replaced with a Löwdin approximation.^378−380^ Instead, in the TDDFT-as method, the latter approximation is not employed and, moreover, the calculation of the semilocal XC kernel contribution on the grid is not required,^383^ as it can be modeled/approximated by the same exponent α of the s-type Gaussian auxiliary basis function. However, the exponent α needs to be optimized for each atom type separately.

**Figure 25:**
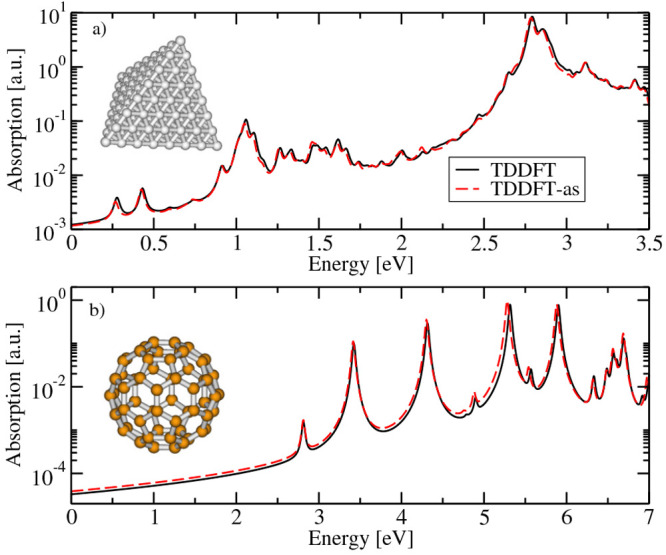
TDDFT and TDDFT-as absorption spectra (in log scale) for (a) Ag_120_ and (b) fullerene determined using the PBE XC functional and a Lorentzian broadening of 20 meV.

Here, we shortly report on results for two computationally expensive cases using the PBE^313^ functional. First, a 120 atom silver nanoparticle, Ag_120_, with *T*_d_ symmetry calculated using the def-SVP^41^ basis set. Second, a fullerene with *C*_1_ symmetry calculated using the def2-TZVP^270^ basis set. Both systems contain only a single type of atom. We optimized α by minimizing the root-mean square (RMS) averaged excitation energy error *E*_avg_, avoiding state flipping,^383^ and considering 400 *t*_2_ (600 *a*) excited states for the silver nanoparticle (fullerene). The optimization yields α_Ag_ = 0.036 with *E*_avg_ = 5 meV and α_C_ = 0.18 with *E*_avg_ = 12 meV for Ag_120_ and C_60_, respectively, as reported in Table 5. The amazing accuracy is also retained when the maximum error on all excitation energies *E*_max_, including many optically dark states in *C*_60_, is considered. This is demonstrated in Table 5 and Figure 25, where we report the absorption spectra of the two systems considered. The TDDFT-as absorption spectra in Figure 25, reported on a log scale to also highlight states with low oscillator strengths, can be hardly distinguished from the reference TDDFT results in a wide energy range for both systems investigated. Compared to TBLR approaches,^383^ the accuracy of TDDFT-as is therefore increased by an order of magnitude, showing that it is a highly competitive approach.

**Table 5:** Optimized Exponent of the s-Type Auxiliary Basis Function, RMS-Averaged (*E*_avg_) and Maximum (*E*_max_) Errors on Excitation Energies, and Computational Cost (on a Single-Core Intel Xeon Gold 6132) for the First Davidson Step for the RI-*J* and XC Part for the Two Systems Considered^a^

	Ag_120_	C_60_
α	0.036	0.18
*E*_avg_, meV	5	12
*E*_max_, meV	11	39
TDDFT RI-*J*, s	5719	7148
TDDFT-as RI-*J*, s	116	104
TDDFT XC, s	22363	29845
TDDFT-as XC, s	0	0

a In the TDDFT(-as) calculations, the 1s core orbital and the 4s4p orbitals were kept frozen in C_60_ and Ag_120_, respectively.

The computational speed up of the TDDFT-as method is shown in Table 5. The XC part is completely neglected, and the RI-Coulomb part is reduced by a factor of 50–70 due to the strongly reduced dimension of the auxiliary basis set.

More recently, TDDFT-as was extended to hybrid functionals and defined across the periodic table, forming the TDDFT-ris model.^384^ Using the PBE0 hybrid functional^344^ on a test set comprising small-to-large organic molecules, TDDFT-ris has an average error of *E*_avg_ = 60 meV^384^ compared to an average error of *E*_avg_ = 240 meV for sTDDFT.^378,379^ Thus, the TDDFT-as and TDDFT-ris methods, both fully available in the next release, are efficient and accurate approximations of standard TDDFT, providing a significantly less empirical alternative to TBLR approaches. Thanks to the flexible and efficient implementation, accurate simulations of the absorption spectra of large nanoparticles and organic molecules are available at a fraction of the computational cost of standard TDDFT.

### 4.4 Multiscale Modeling Extensions for the Nonlinear Optical Response of Molecular Materials

Further ongoing work concerning multiscale modeling of optical molecular materials is envisaged in the direction of integrating the automatic construction of T-matrices, as used in the multiscale approach in section 3.9.1. These T-matrices or effective material parameters derived thereof are often used in scattering codes and finite-element method-based programs. Examples for such codes are the multilayered periodic general Mie method code (mpGMM), JCMsuite, and COMSOL Multiphysics, which describe light–matter interactions of complex optical devices made form novel materials. In the foreseeable future, the current developments and implementations will be converted into a fully fledged workflow for optical material simulations. This will make the *ab initio*-based T-matrix approach available to a broader scientific community interested in a bottom-up approach of simulating complex artificial molecular materials and photonic devices. Future work will be dedicated to nonlinear optical properties, as this topic is currently seeing increasing interest in the scientific community. The change in the molecular dipole moment *Δμ*_*i*_ (polarization) upon exposure to the oscillating external electric field *E*_*i*_ at the excitation frequencies is often expressed as a power series of the incident field *E*.
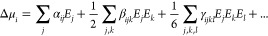
12

In eq 12, α_*ij*_ denotes the polarizability, β_*ijk*_ denotes the first hyperpolarizability, γ_*ijkl*_ denotes the second hyperpolarizability and so on. Currently, optical multiscale studies are limited to linear response, taking into account α_*ij*_.^254,261^ To take into account nonlinear effects, *i.e.*, β_*ijk*_ and/or γ_*ijkl*_, the additionally arising quadratic (and/or cubic) response terms of eq 12 need to be taken into account. While TURBOMOLE already allows calculation of the first hyperpolarizabilities β_*ijk*_ for real frequencies, ongoing work is dedicated to expanding this toward general complex frequencies. Ultimately, this will allow not only studies of the nonlinear light–matter interactions on the individual molecular level but also the construction of “hyper-T-matrices”. The latter can be used to investigate for example second-harmonic generation (SHG) efficiencies, macroscopic second-order susceptibilities, and two-photon absorption of photonic devices made from molecular materials.

### 4.5 Relativistic Effects and Magnetic Properties of Periodic Systems

As is evident from the many sections in this Review focusing on molecules, TURBOMOLE was initially developed to study finite molecular systems. However, the code infrastructure was extended to support calculations with periodic boundary conditions almost 15 years ago,^55^ and developments for molecular systems can be transferred to the periodic code. Recently, a two-component DFT framework was implemented for ground-state calculations, supporting energies and various plotting options for bands and the electron density on a grid.^385^ The reason to use such a framework for periodic systems is twofold. First, it allows for the inclusion of spin–orbit coupling in a variational ansatz for relativistic effects. Second, the 2c formalism is necessary to study magnetic properties and arbitrary spin alignments, *i.e.*, ferromagnetic, antiferromagnetic, and noncollinear spin textures.

A pilot application to the band gaps of AgI is shown in Table 6. Here, relativistic effects substantially affect the band energies. That is, the nonrelativistic approach shows a large deviation toward the four-component (4c) ansatz directly based on the Dirac equation. In contrast, the scalar 1c approach yields substantial improvement. The spin–orbit 2c approach, employing ECPs, further improves upon these results and is in good agreement with the four-component reference. This shows that the 2c framework serves as an excellent approximation with drastically reduced computational demands. Here, a Kramers-restricted (time-reversal symmetric) approach is available for closed-shell systems, whereas a Kramers-unrestricted approach (breakdown of time-reversal symmetry) is used for open-shell systems. For the latter, a noncollinear treatment of the electron spin is applied.

**Table 6:** Energy Band Gaps (in eV) of Three-Dimensional AgI (Lattice Constant 6.169 Å, Rocksalt Structure^386^) Obtained for Various *k*-Points with the PBE Functional^a^

method	L–L	Γ–Γ	X–X	L–X
NR	3.89	3.42	3.71	1.48
1c ECP	3.49	2.16	2.98	0.65
2c ECP	3.25	1.82	2.69	0.41
4c DKS	3.25	1.88	2.74	0.49

a Nonrelativistic calculations (NR) are performed with the TZVPalls2/TZVPall basis set,^387^ whereas the ECP-based 1c and 2c calculations use the dhf-SVP(-2c) bases.^388^ Results taken from ref (385). Four-component Dirac–Kohn–Sham (DKS) reference values are taken from ref (389), employing the uncontracted Dyall-VDZ basis.^390,391^.

Current endeavors cover the extension of the 2c formalism to energy gradients^56^ and the stress tensor,^59^ as well as the inclusion of the current density,^151^ see section 3.2. This will allow us to perform structure optimizations and sophisticated studies with τ-dependent functionals, as the current density is of crucial importance for materials such as Weyl semimetals^392^ and magnetic Hopfions.^393^ Another strong point of the 1c implementation for periodic systems is the availability of HF exchange, which can be applied at a reasonable cost as localized basis functions are used.^328^ This allows for the use of generally all available global and range-separated hybrid functionals in a stable and convergent framework. Extension of this feature to the 2c framework will allow for a more precise description of band gaps and other properties. For example, for magnetic properties it was shown that the amount of HF exchange incorporated is crucial.^119^ Therefore, a robust implementation of 2c HF exchange in the periodic framework will be useful in determining related quantities also for materials in the solid state and nanostructures.

## 5 Outlook

The quality of a code strongly correlates with the health and functioning of its developers’ community.^394^ TURBOMOLE developers are organized in small units pursuing their own scientific agendas, as illustrated by this Review. While this is a typical and, to a degree, necessary *modus operandi* for large scientific coding projects, the need to secure original authorship and demonstrate scientific independence often conflicts with sharing plans and code, taking collective responsibility, and avoiding “technical debt”. As a result, TURBOMOLE has historically not been particularly easy to use, contribute to, or interface with other codes. TURBOMOLE GmbH was founded precisely to address these issues and has provided a framework to advance common goals and improve code quality. Nevertheless, incentives to collaborate and adopt sustainable coding practices remain few and far between. The future of the TURBOMOLE project will vitally depend on whether the conditions set by the environment, *i.e.*, academic institutions, funding agencies, reviewers, the developers, and not least the users, foster a thriving and collaborative community, which incentivizes continued investment in the code base.
